# Chromatin accessibility derived from cfDNA serves as a novel classification biomarker of glioma

**DOI:** 10.3389/fonc.2025.1688625

**Published:** 2025-12-15

**Authors:** Qiuyue Wu, Jian Wu, Fan Wang, Qiang Wang, Wei Dai, Yang Yang, Chuanyue Zhang, Ying Han, Min Chen, Hao Pan, Lan Luo, Xinyi Xia

**Affiliations:** 1 Institute of Laboratory Medicine, Jinling Hospital, Affiliated Hospital of Medical School, School of Life Sciences, Nanjing University, Nanjing, Jiangsu, China; 2 Department of Bioinformatics, Nanjing Medical University, Nanjing, China; 3 Institute of Laboratory Medicine, Jinling Hospital, Affiliated Hospital of Medical School, Nanjing University, Nanjing, Jiangsu, China; 4 Department of Neurosurgery, Jinling Hospital, Affiliated Hospital of Medical School, Nanjing University, Nanjing, Jiangsu, China; 5 School of Animal Science and Food Engineering, Jinling Institute of Technology, Nanjing, China; 6 State Key Laboratory of Analytical Chemistry for Life Science, Nanjing University, Nanjing, Jiangsu, China; 7 Jinling Hospital, The First School of Clinical Medicine, Southern Medical University, Nanjing, Jiangsu, China

**Keywords:** glioma, cell-free DNA, chromatin accessibility, liquid biopsy, cerebrospinal fluid, plasma

## Abstract

**Introduction:**

Gliomas can be classified by their molecular characteristics, which are also closely associated with clinical outcomes. Cell-free DNA (cfDNA)-based liquid biopsy in gliomas is challenging because of the limited amount of tumor-derived cfDNA present in body fluids.

**Methods:**

In this study, we identified the open chromatin states of gliomas using cfDNA and demonstrated the potential of this technique for glioma detection. The chromatin accessibility of gliomas was investigated using tumor tissues donated by four donors. cfDNA derived from paired cerebrospinal fluid (CSF) and plasma samples was also sequenced.

**Results:**

A total of 72 accessible chromatin regions in the tumor tissues were identified as open chromatin regions using CSF cfDNA. Furthermore, 16 open chromatin regions with significant differences in glioma grade were found using cfDNA extracted from plasma. A glioma grade classifier was constructed with 16 plasma cfDNA-derived accessible chromatin features, which could accurately differentiate low-grade from high-grade samples in the training dataset Area Under Curve ((AUC) = 0.814). However, lower accuracy was obtained on the testing dataset (AUC = 0.736). The diversity of transcription factor binding sites among glioma patients was also illustrated using cfDNA.

**Discussion:**

In conclusion, our study defines novel chromatin-accessibility-based biomarkers and illustrates their potential application in glioma liquid biopsy.

## Highlights

- cfDNA derived from glioma patients could be used to decode chromatin accessibility.
- Epigenetic-based cfDNA marker displayed potential in differentiating glioma patients.
- Chromatin accessibility analysis has opened up potential clinical utilities of cfDNA.

## 1 Introduction

Glioma, as the most common malignancy of the brain, still has a high mortality rate (1). The prognosis for patients with glioblastoma is poor, with a median survival of less than 2 years despite optimal multimodal therapy, such as incorporating surgery, radiotherapy, systemic therapy (chemotherapy and targeted therapy), and supportive care (2). The optimal treatment for glioma strongly depends on correctly identifying the patient’s subtype (3). An accurate diagnosis is vital for the treatment of glioma patients and greatly influences the patient’s outcome (4). In recent decades, significant advancements have been achieved in the molecular profiling of glioma; molecular markers, such as IDH mutation and 1p/19q co-deleted, have been identified as key targets for the diagnosis of gliomas (4). The diagnosis of molecular features in glioma patients is mainly based on the analysis of tumor tissue (5). However, performing a biopsy or resection for diagnostic purposes can carry disproportionate risk, especially for glioma patients with tumors in sensitive locations (6). In addition, patients with recurrent or progressive brain tumors may not wish to undergo another biopsy to evaluate tumor status, even though these treatments may identify new therapy targets and improve prognosis.

Liquid biopsy provides exciting opportunities to identify the molecular landscape of tumors in a non-invasive way. Liquid biopsy has been widely used in several types of cancer to classify subtypes and monitor disease progression and response to therapy, especially for extracerebral tumors (7). Due to its non-invasive nature, plasma-based cell-free DNA (cfDNA) detection is the main method used in liquid biopsy. However, due to the blood–brain barrier (BBB), a low amount of circulating tumor DNA (ctDNA) is found in plasma derived from glioma patients (8). Compared with plasma, cerebrospinal fluid (CSF) has been shown to contain a higher fraction of cfDNA derived from carcinoma in patients with brain tumors and has been suggested as a superior source of ctDNA (9, 10). For this reason, various methods have been used to analyze the cfDNA in CSF, including targeted sequencing of known driver mutations (11), methylation profiling (12), and copy number variation detection (3), to accelerate the diagnosis and treatment of brain tumors. These methods of liquid biopsy for brain tumors mainly target tumor tissue-derived DNA, which is found in only 49.4% of patients with glioma (13). This suggests that the limited detection of this specific DNA in CSF may be due to these reasons. For the detection of driver mutations, some mutations may be missing owing to the lack of ctDNA in CSF. In addition, although cerebrospinal fluid has low intrinsic nuclease activity (14), ctDNA containing the target mutations has great potential for degeneration. Furthermore, *de novo* glioblastoma (GBM) harbors a lower genetic alteration burden compared with other cancers (15), which presents a natural obstacle to these methods. The challenge of detecting ctDNA-based methylation or copy number variations (CNV) is the low ratio of patients with CSF ctDNA. The methylation state of cfDNA has great potential to overcome the shortcomings of genetic features, but the lengthy steps of DNA methylation detection can cause the loss of DNA and reduce detection sensitivity.

Apart from the role of genetics in cancer progression, chromatin accessibility is another important epigenetic feature essential for regulating the expression of genes. Disruptions to accessibility have been implicated in driving cancer initiation, progression, and metastasis (16). Glioma, as a therapy-resistant cancer, exhibits a high level of heterogeneity among patients, within tumors, and in the tumor microenvironment. The epigenetic heterogeneity, especially in glioblastoma, plays an important role in the development, progression, and prognosis of patients (17). The chromatin landscape differs significantly between primary and recurrent glioblastoma tumors, with recurrent tumors shifting to a mesenchymal phenotype with distinct chromatin accessibility features (18). The diversity of chromatin accessibility features of glioma patients provides an opportunity to diagnose the subtype of glioma, track the evolution of the disease, and monitor the therapeutic effects in patients using a liquid biopsy method. The chromatin accessibility is closely related to nucleosome positioning, which could greatly influence the characteristics and distribution of cfDNA. For this reason, several methods based on this conception were developed to decode the epigenetic information contained in cfDNA, such as DNA evaluation of fragments for early interception (DELFI) (19), window protection score (WPS) (20), and promoter fragmentation entropy (PFE) (21). In addition, the feature of fragmentomics is also closely related to chromatin accessibility (22).

To utilize the feature of chromatin accessibility as a powerful marker in liquid biopsy for glioma, the open chromatin state of glioma was investigated using tumor tissues and cfDNA derived from paired CSF and plasma in this study. A total of 72 genome regions were identified as open chromatin in both tissue and CSF cfDNA samples. Within these genome regions, low-grade glioma (LGG)- and GBM-specific open chromatin regions were identified. LGG patients, classified by the expression of three genes related to LGG-specific open genome regions, showed significant differences in survival. It was also found that 311 CSF cfDNA-derived genome regions were significantly different between LGG and GBM patients. To further extend the usage of the chromatin accessibility feature, 16 genome regions that were significantly different in plasma cfDNA-derived open chromatin states were identified and used to construct a classifier to distinguish the type of glioma in patients using plasma cfDNA.

## 2 Materials and methods

### 2.1 Patients and clinical data

Fresh resected tumor tissues, cerebrospinal fluid, and whole blood were collected with informed consent from four patients who were enrolled at Jinglin Hospital (Nanjing, China) without preoperative treatment. The WHO grades of the patients included in our study were classified by histopathology combined with molecular pathology. This study of human specimen collection was approved by the Ethics Committee of Jinglin Hospital. The clinical information is available in **Supplementary Table 1**.

### 2.2 Tumor dissociation

Fresh resected tissues were minced, rinsed with phosphate-buffered saline (PBS; Thermo Fisher Scientific, USA), and incubated in digestion buffer (KeyGEN, KGA3104-100) in a water bath at 37 °C. During digestion, the tissues were gently mixed every 5 min to improve the digestion efficiency until most cell clusters were in suspension. The mixture was centrifuged at 1,000 rpm for 10 min and washed once with 5 mL of PBS, and the precipitate was resuspended in 1 mL of PBS. The cell density was counted using a hemocytometer and diluted to a suitable density with PBS.

### 2.3 Preparation of plasma and CSF

The whole blood was collected in an ethylenediaminetetraacetic acid (EDTA) anticoagulation tube, and the CSF was collected in sterile specimen tubes before surgery. Both plasma and CSF were treated immediately once they were collected. To separate the plasma, whole blood was spun at 1,600 *g* for 15 min at 4 °C, and then the supernatant was transferred to a new centrifuge tube. The tube containing the supernatant was spun at 16,000 *g* for 10 min at 4 °C, and the supernatant was collected. All plasma was stored in Eppendorf tubes at −80 °C until use. The CSF was treated following the same procedure as that for the plasma and stored at −80 °C before use.

### 2.4 Preparation of various adaptors

The Single Strand Adaptor Library Preparation (SALP) method was used to investigate the chromatin accessibility of tumor tissues (23). The adaptors were prepared following the procedure described in our previous paper (23). Briefly, oligonucleotides were all synthesized by Sangong Biotech (Shanghai, China) (**Supplementary Table 2**). Barcode and mosaic end (ME) oligos were dissolved in ddH_2_O to a final concentration of 20 μM and then mixed in equimolar amounts in a PCR tube to generate barcoded Tn5 adaptors (BTAs). In order to prepare single-strand adaptors (SSAs), SSA-PN and SSA-PNrev oligos were dissolved in ddH_2_O at a concentration of 20 μM and then mixed in equimolar amounts in a PCR tube. All oligo mixtures were denatured in a water bath at 95 °C for 5 min and then gradually cooled to 25 °C for annealing.

### 2.5 Preparation of barcoded Tn5 transposome

Following the instructions for Tn5 transposase (Robust Tn5 Transposase, Robustnique Corporation Ltd., Tianjing, China), 4 μL of BTA (10 μM) was mixed with 2 μL of 10× Tn5 transposome assembly buffer (TPS), 1 μL of Tn5 transposase, and 13 μL of H_2_O to generate a 20-μL reaction volume. The final reaction was gently mixed and incubated at 25°C for 30 min to generate the Tn5 transposome.

### 2.6 Tagmentation of chromatin from tumor tissues

Tagmentation was performed using 100,000 cells collected from four tumor tissues. Cells were collected by spinning at 500 g for 5 min at 4°C and then washed once with 50 μL of cold PBS. The cells were lysed by resuspending them in cold lysis buffer (10 mM Tris-HCl, pH 7.4, 10 mM NaCl, 3 mM MgCl_2_, and 0.1% IGEPAL CA-630). The cells were then spun at 500 g for 10 min at 4°C to collect the nuclei precipitate. For tagmentation, the nuclei were tagmented in a 30-μL reaction containing 20 μL of Tn5 transposome, 3 μL of Dimethylformamide (DMF), and 1× Tn5 transposome reaction buffer (LM buffer). The tagmentation reactions were mixed gently and incubated at 37°C for 30 min, with gentle mixing every 10 min to improve tagmentation efficiency. In tagmentation, different BTAs (**Supplementary Table 2**) were used to tagment the different cell samples. After tagmentation, the tagmented chromatin from different patients was pooled together. The chromatin mixture was incubated at 65°C for 1 h with 0.1% Sodium dodecyl sulfate (SDS) and 400 μg/mL Proteinase K (Sigma, Germany). After incubation, the DNA was purified with standard phenol/chloroform extraction.

### 2.7 Preparation of SALP-seq libraries with tagmented chromatin of various samples

To prepare the SALP library, the purified gDNAs were denatured at 95°C for 5 min and chilled on ice immediately for 5 min. Then, the denatured gDNAs were ligated with SSA in a 10-μL reaction volume with 1 μL of T4 DNA ligase (NEB, USA, M0202L), 1× T4 DNA ligase buffer, and 1 μL of SSA (5 μM) at 16°C overnight. Then, the reaction was mixed with an equal volume of 2× rTaq mix (Takara, Kusatsu, Japan) and incubated at 72°C for 15 min. The SSA-linked gDNAs were purified with 1.2× Ampure XP beads (Beckman Coulter, Beckman Coulter, California, USA) and then amplified with selected index primers in a 50-μL PCR reaction containing 25 μL of NEBNext^®^ Q5^®^ Hot Start HiFi PCR Master Mix (NEB, M0543S), 1 μL of NEBNext Universal PCR Primer (10 μM), and 1 μL of NEBNext Index Primers (10 μM). The PCR program was as follows: i) 98°C for 5 min; ii) 98°C for 10 s, 65°C for 30 s, and 72°C for 1 min, 18 cycles; and iii) 72°C for 5 min. The PCR products were run on an agarose gel, and DNA fragments of 300–1,000 bp were extracted using the QIAquick Gel Extraction Kit.

### 2.8 cfDNA extraction and NGS library construction

The extraction of CSF and plasma cfDNA was performed according to the instructions for QIAseq cfDNA All-in-One Kits (QIAGEN, Germany, 180023). A volume of 1.5 mL of CSF or plasma was used to extract the cfDNA. All cfDNA extracts from 1.5-mL samples were used to construct the sequencing libraries. The sequencing libraries were also constructed following the manufacturer’s instructions for QIAseq cfDNA All-in-One Kits (QIAGEN, 180023). Briefly, the library preparation procedure included end-polishing and adaptor ligation. All adaptor ligation products were amplified as follows: i) 98°C for 2 min; ii) 98°C for 30 s, 60°C for 30 s, and 72°C for 30 min, 10 cycles; and iii) 72°C for 1 min. Then, the constructed sequencing libraries were pooled to generate the final sequencing library.

### 2.9 NGS

All next-generation sequencing (NGS) libraries were quantified using Qubit and quality checked using Agilent Bioanalyzer 2100 (Agilent, USA). All libraries were sequenced using the Illumina HiSeq X Ten platform (Nanjing Geneseeq, Nanjing, China).

### 2.10 Sequencing data analysis

To investigate chromatin accessibility using SALP-seq, the raw read data were separated according to the index and barcode using homemade Perl scripts. Then, the ME (19 bp) and barcode (6 bp) sequences were removed from the 5' end of the paired-end sequencing read 2. The quality control of fastqs was performed using fastp. Clean reads were aligned to the human genome (hg38) with Bowtie2 (version 2.4.5) using the default settings, except that the -X parameter was set as 2,000 to ensure that the long fragments could be aligned to the genome (24). Peak calling was performed using macs2, with the following parameters: –shift -75 –extsize 150 –nomodel –call-summits –nolambda –keep-dup all -p 0.01. The peak sets were generated as described by Corces et al. (25). Briefly, the peak summits were extended by 250 bp on either side to a width of 501 bp. Peaks that overlapped with the hg38 blacklist were removed. After that, the most significant peak was kept, and the overlapped peaks were removed. Then, this process was repeated until all peaks were the most significant peaks or removed due to overlap with significant peaks. Peak annotation and functional enrichment analysis were performed using the ChIPseeker package (26). Differential analysis was performed using the limma package. Intersection analysis was performed using the bedtools intersect function (27).

To analyze the Assay for Transposase-Accessible Chromatin using sequencing (ATAC-seq) data, the raw sra data were transferred to fastq using the fastq-dump program with the –split-3 parameter. The reads were aligned to the hg38 reference genome using Bowtie2, and then the ROSE program (28) was used to identify the super-enhancer. The chromatin immunoprecipitation sequencing (ChIP-seq) datasets were analyzed using the same parameters as ATAC-seq data in the sra transform and aligned to the reference genome steps. The genome coverage of ChIP-seq data was calculated using the bedtools bamCoverage function, and then custom tracks were constructed and shown in the UCSC Genome Browser. The transcription factor binding feature was analyzed using MEME suite (29) with HOCOMOCO 13 core as a reference.

To define chromatin accessibility features using CSF cfDNA, 500-bp genome regions located upstream and downstream of the common genome open regions identified in tissue samples were designated as Peak-F (500-bp genome region upstream peak) and Peak-B (500-bp genome region downstream peak), respectively. The CSF cfDNA read counts in the three kinds of peaks were calculated. Then, significant differences between Peak-F, Peak, and Peak-B were identified using the Wilcoxon test and identified as open chromatin regions based on the CSF cfDNA. To investigate the reliability of the open chromatin regions overlapped between tumor tissue and CSF cfDNA samples, the hg38 reference genome was divided into 500-bp windows. Then, genome regions that overlapped with open chromatin regions identified with tissues were removed to generate a background. Fisher’s exact test was performed to identify the significance of the overlap.

To compare the read distribution between open chromatin regions and cancer-related mutation-containing genome regions, well-known cancer-related mutations were collected from the MSK-IMPACT gene panel; 90-bp genome regions located upstream and downstream of the mutations were generated to construct a genome region set with similar length of cfDNA fragments. Then, cfDNA read counts of each genome region in all samples were calculated using the featureCount program. The significant statistical analysis was performed using the Wilcoxon test in R.

The survival R package was used to perform survival analysis using bulk RNA-seq datasets and clinical information from The Cancer Genome Atlas (TCGA), downloaded through UCSC Xena. The scores for each patient were calculated using the single-sample gene set enrichment analysis (ssGSEA) algorithm from the Gene set variation analysis (GSVA) package based on the selected genes (30). The samples were divided into high signature score and low signature score groups using the median value of the calculated scores. To construct the glioma grade classifier using genomic features, public plasma cfDNA 5hmC sequencing data (accession number: GSE132118) were obtained from Gene Expression Omnibus (GEO) (http://www.ncbi.nlm.nih.gov/geo), which contained 111 samples (WHO grade II, 32 samples; WHO grade III, 15 samples; WHO grade IV, 64 samples). The raw data were mapped to the hg38 reference genome using Bowtie2 with default parameters. Then, read counts in each identified genome region were calculated using the featureCount program to construct a count matrix for building a classifier. Glioma samples classified as grades II and III were identified as low-grade, and grade IV samples were identified as high-grade. Then, a total of 111 samples were randomly divided into training and testing datasets at a ratio of 7:3. An XGBoost model was used to construct the classifier with the XGBoost package (31), and 10-fold cross-validation was performed to increase the reliability of the classifier.

## 3 Results

### 3.1 Differential chromatin accessibility identified from tumor tissues among glioma patients

**Figure 1:**
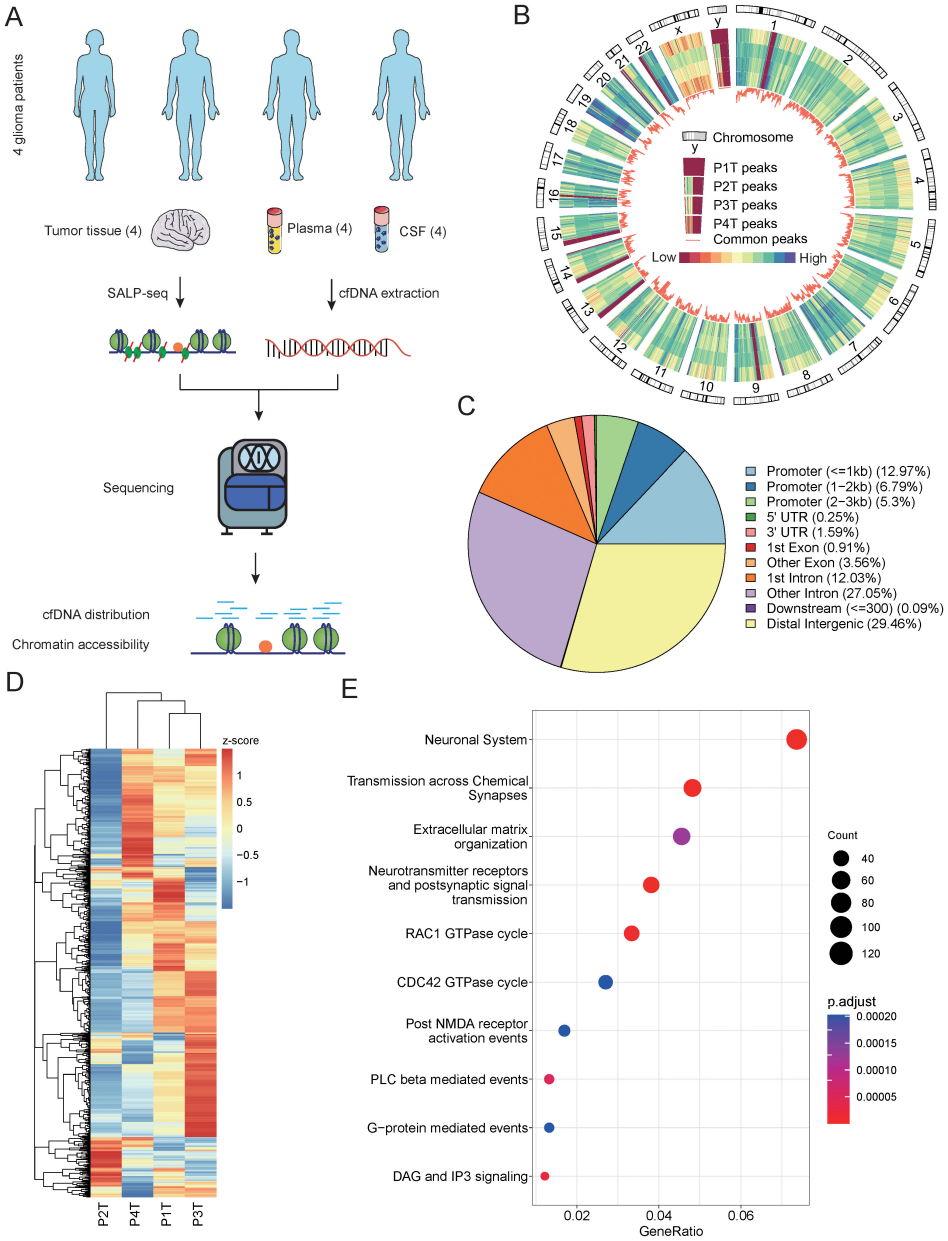
Chromatin accessibility identified from glioma tumor tissues. **(A)** Schematic overview and the number of samples of this study. The open chromatin state was explored and compared among different glioma patients using tumor tissues and cfDNA. **(B)** The distribution feature of open chromatin regions in four glioma patients. A close-up of chromosome Y is provided to illustrate the order of samples. The color bar represents values from low to high. P1T to P4T represent the tissues from patient 1 to patient 4. **(C)** The genomic distribution of accessible chromatin regions identified using tissue samples from four glioma patients. The genomic features of the open chromatin regions are annotated, and the percentages of each genomic feature are calculated. **(D)** A comparison of the read number in the top 1,000 open chromatin regions of the four glioma patient tissue samples. **(E)** The top 10 Kyoto Encyclopedia of Genes and Genomes (KEGG) pathways enriched by the open chromatin regions identified in the four tissue samples. cfDNA, cell-free DNA.

To explore the chromatin accessibility of gliomas and identify the potential relationship between tumor tissues and cfDNA, we designed a workflow to compare the open chromatin states identified from tissues and cfDNA (**Figure 1A**). To investigate the diversity of chromatin accessibility between glioma patients, we performed Tn5-based SALP-seq on four glioma tumor tissues (**Supplementary Figure 1A**). As described in our previous paper (23), the Tn5-based SALP was based on a kind of specific design adaptor. During the Tn5 dimmer construction procedure, we used only one kind of adaptor, which could overcome the shortage of ATAC-seq. Based on this feature, Tn5-based SALP-seq has the potential to capture more open chromatin regions, especially the genome regions with lower chromatin accessibility. The distribution of open chromatin regions identified in each tissue sample was distinct (**Figure 1B**). The genome position of the common open chromatin regions in four samples was closely related to the coefficient of variation (CV) of the peak number (**Supplementary Figure 2A**). Genome regions with a lower CV contained a high density of common open chromatin regions, while regions with a high CV exhibited diverse chromatin accessibility features (**Figure 1B**, **Supplementary Figure 2A**). The diversity of open chromatin states was also distinct among different chromosomes. Apart from the sex chromosomes, chromosome 18 showed the highest CV (**Supplementary Figure 2B**). Further analysis of the genome regions with open chromatin in the four samples showed heterogeneity among the patients, especially in promoter regions (**Supplementary Figure 2C**). We identified a total of 12,066 peaks as shared among different patients, with these open chromatin regions mainly located in distal intergenic areas and introns (**Figure 1C**). The read counts of the common open regions also varied among different samples (**Figure 1D**). In each sample, distinct genome regions with a high read density could be found (**Figure 1D**), suggesting that they play important roles in the heterogeneity of glioma. To investigate the role of these common open genome regions, we performed pathway annotation. Several glioma-related pathways were enriched based on these common open chromatin regions (**Figure 1E**), such as the neuronal system, transmission across chemical synapses, and neurotransmitter receptors and postsynaptic signal transmission. To further investigate the diversity of chromatin accessibility of genome regions, we evaluated the open chromatin state using public ATAC-seq datasets. Based on chromatin accessibility identified using cell lines, we observed high diversity in both GBM patient-derived cell lines and astrocyte cell lines (**Supplementary Figure 3A**). The high diversity of chromatin accessibility was also demonstrated using ATAC-seq derived from GBM organoid samples (**Supplementary Figures 3B, C**). There were also 540 genome regions with significantly different chromatin accessibility between GBM organoid and paired margin samples (p value < 0.05, log2 fold change >|2|) (**Supplementary Figure 3D**). These results illustrate the chromatin accessibility diversity of glioma patients, providing a foundation for detecting the specific features of the open chromatin state in each patient using cfDNA.

### 3.2 Chromatin accessibility diversity contained in CSF cfDNA

**Figure 2:**
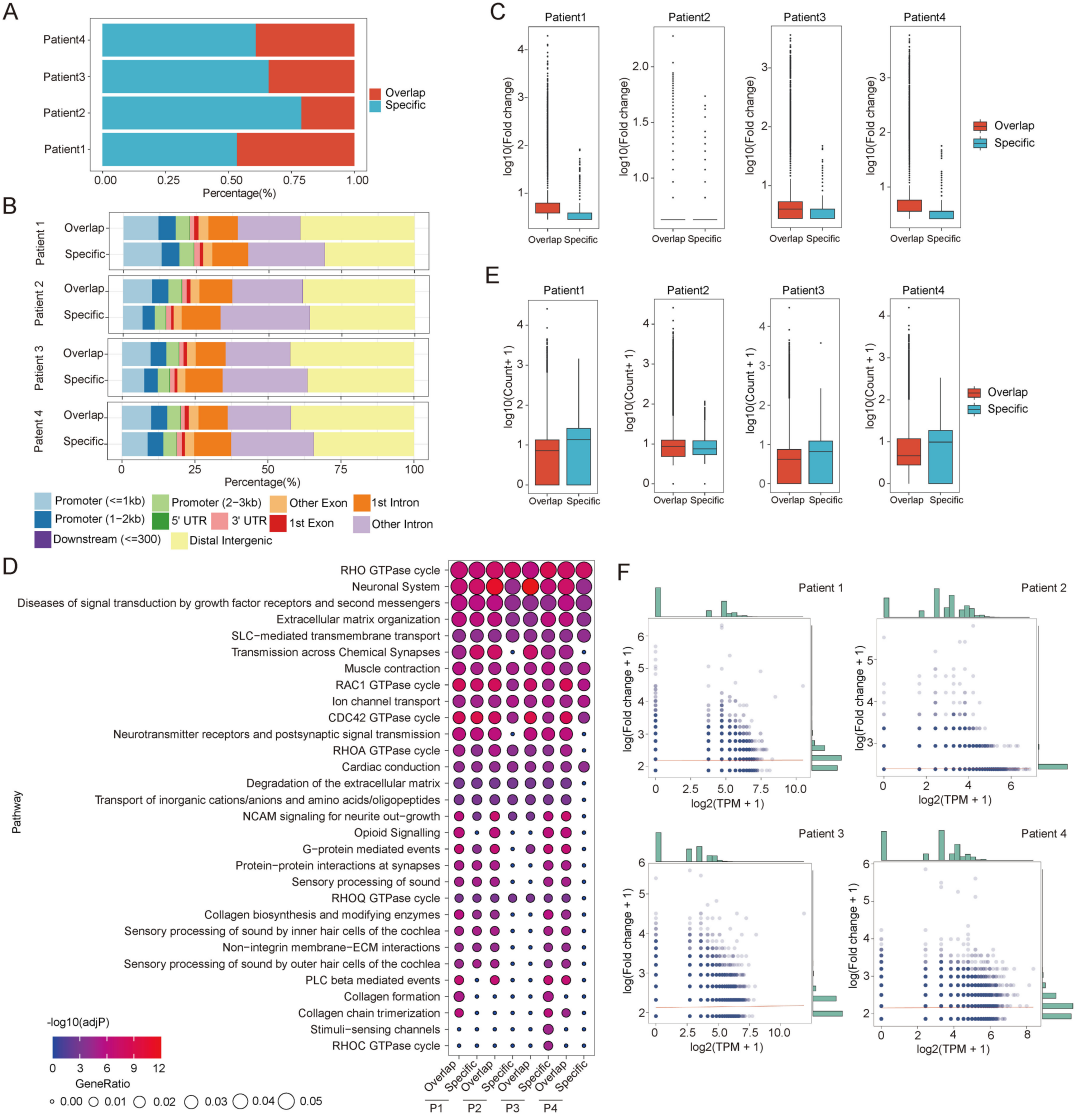
The diversity of CSF cfDNA-identified open chromatin regions in glioma patients. **(A)** The composition of accessible chromatin regions in each patient. The common and specific open chromatin regions were compared among the patients. Common represents the open chromatin genome regions common with other patients. Specific represents the specific open chromatin regions identified in each patient. **(B)** The genomic features of different types of open chromatin regions in each patient. **(C)** A comparison of the read count in different types of open chromatin regions. The colored boxplots show the logged read counts for the two groups of genome regions identified in panel **(A, D)** A comparison of the fold changes of different types of open chromatin regions. The colored boxplots show the logged read counts for the two groups of genome regions identified in panel **(A, E)** Enriched pathways in two types of open chromatin regions of each patient. The colors of the points indicate the adj-p-value of the pathway in patients. The size of the points indicates the gene ratio of each enriched pathway. **(F)** The relationship between normalized read counts and fold changes for open chromatin regions in each patient. CSF, cerebrospinal fluid; cfDNA, cell-free DNA.

The high diversity of chromatin accessibility features among different glioma patients could be beneficial for their use as liquid biopsy markers. To investigate the chromatin feature encoded in cfDNA, we performed high-throughput sequencing with tissue paired CSF and plasma cfDNA (**Supplementary Figures 1B, C**). We performed a further comparison of the peaks identified using tissue samples from four glioma donors. First, the peaks of each sample intersected. We identified overlapping and specific peaks for each sample (**Figure 2A**). Similar to our previous results, the open chromatin state was distinct among the patients, as reflected by the percentage of overlapping peaks for each patient. All patients exhibited over 50% specific peaks, and in patient 2, the ratio was over 75% (**Figure 2A**). The genomic features of overlapping and specific peaks were also different in each patient. The majority of specific peaks in three patients were located in non-promoter regions compared with the overlapping peaks, which indicates that these peaks have a distal regulation function (**Figure 2B**). Meanwhile, in patient 1, the proportion of specific peaks located in promoter regions was higher than that of overlapping peaks (**Figure 2B**). These results confirm the high chromatin accessibility diversity among glioma patients. The open chromatin level is closely related to gene transcription regulation, and different open state levels also play important roles in the heterogeneity of glioma by providing binding sites to transcription factors. For this reason, we compared the fold changes of overlapping and specific peaks among different patients. The fold changes in overlapping peaks were greater than those of specific peaks in all patients, indicating that the overlapping peaks exhibited higher open chromatin levels (**Figure 2C**). Based on these results, we inferred that the low open chromatin level of patient-specific peaks could result from binding with a low number of transcription factors, which may regulate the specific pathways and contribute to the diversity of glioma. To verify this inference, we analyzed the relationships of the two kinds of peaks with gene regulation pathways. We found that two types of peaks were all taking part in the fundamental cancer pathways in glioma, including the RHO GTPase cycle and neuronal system, which were enriched in all patients (**Figure 2D**). However, the pathways enriched by specific peaks varied among patients. For example, the stimuli-sensing channels and RHOC GTPase cycle were only enriched in patient 3 (**Figure 2D**). In the overlapping peaks, common pathways accounted for a larger part compared to those in the specific peaks (**Figure 2D**). These results further validate the distinct chromatin features among the glioma patients. To verify whether the diversity of chromatin features could be reflected using CSF cfDNA, we compared the cfDNA read distribution between the two kinds of peaks. As a key link between cfDNA and chromatin accessibility, the cfDNA distribution features of overlapping and specific peaks also showed significant differences (**Figure 2E**). Interestingly, the cfDNA distribution trends also varied among the different patients. The cfDNA fragment distributed in the specific peaks of patients 1, 3, and 4 was higher than in the overlapping peaks, but the opposite trend was found in patient 2 (**Figure 2E**). Based on the mechanism of cfDNA generation, we hypothesize that the cfDNA fragment distribution may be closely related to the open chromatin level. To validate this hypothesis, we investigated the relationship between the open chromatin levels of the patient-specific peaks and the cfDNA fragment distribution. All samples exhibited a section of patient-specific peaks that did not contain CSF cfDNA fragments (**Figure 2F**). These results indicate that only some chromatin features can be detected based on cfDNA. The patient-specific peaks containing fewer cfDNA fragments exhibited lower open chromatin levels, which is consistent with our previous conclusion (**Figure 2F**). In addition, we observed a trend where peaks with higher fold changes contain more cfDNA fragments in patients 1, 3, and 4 (**Figure 2F**). Therefore, we inferred that there may be more transcription factor binding events in these regions. Altogether, these results further confirm the diversity of chromatin accessibility in glioma patients and demonstrate that differential open chromatin state can also be detected using CSF cfDNA.

### 3.3 Chromatin accessibility features identified using CSF cfDNA

**Figure 3:**
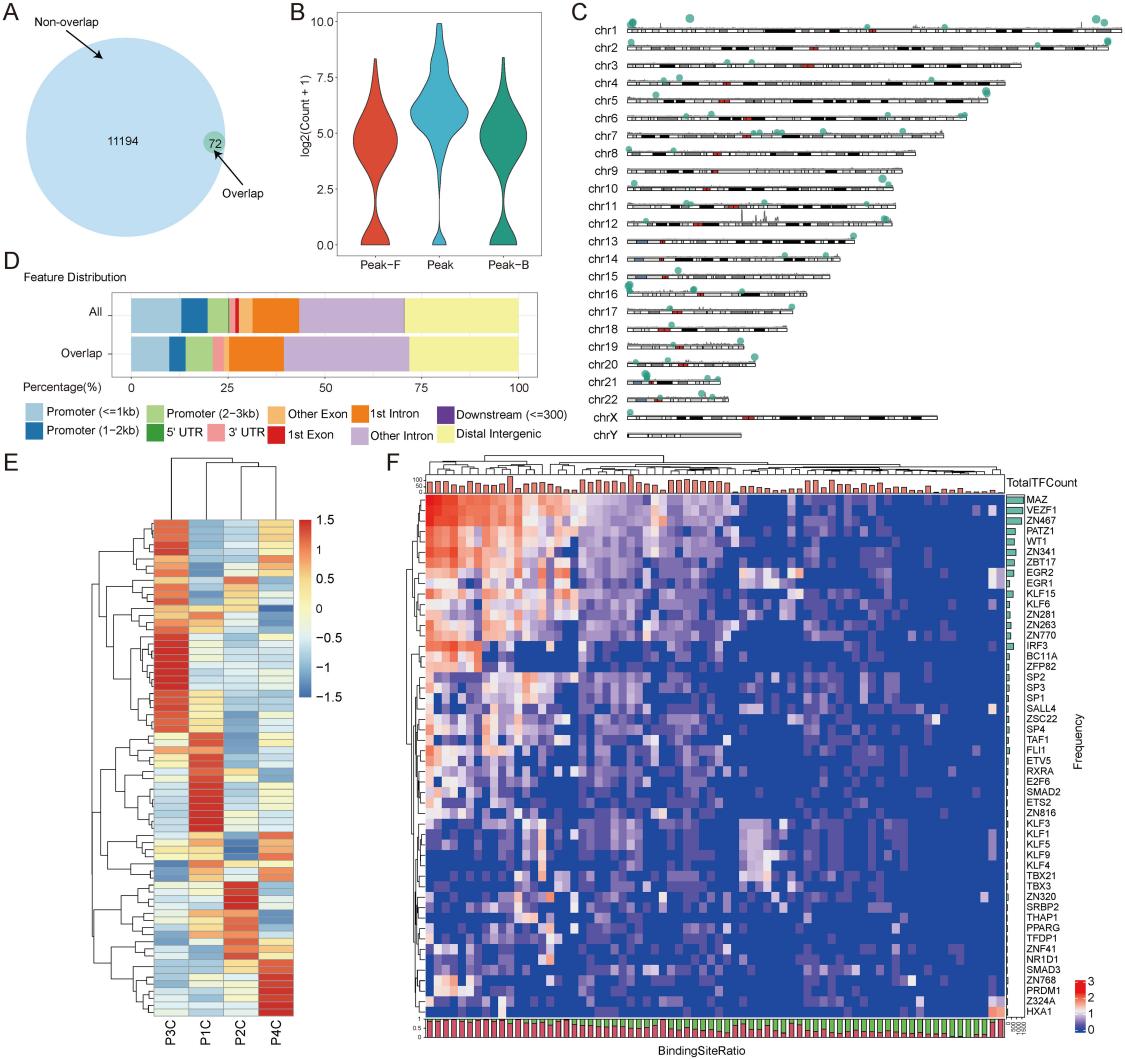
The open chromatin states of glioma patients derived from CSF. **(A)** A Venn diagram displaying the intersections of open chromatin regions identified from CSF cfDNA and tumor tissues. Overlap represents the open chromatin regions identified in both CSF cfDNA and tissues. Non-overlap represents the open chromatin regions identified only in tissues. **(B)** The read counts around the CSF cfDNA-identified open chromatin regions. Peak F represents the 500-bp genome region upstream of the open chromatin region. Peak represents the CSF cfDNA-identified open chromatin region. Peak B represents the 500-bp genome region downstream of the open chromatin region. **(C)** The distribution of the CSF cfDNA-identified open chromatin genome regions throughout the genome. A histogram showing the density of the open chromatin regions identified from tissue samples in 1-MB windows. The locations of the bubbles indicate the positions of the open chromatin regions identified from CSF cfDNA, and the size of the bubbles represents the peak scores calculated from the tissue samples. **(D)** Comparison of genomic distributions between all tissue-identified open chromatin regions and all CSF cfDNA-identified accessible chromatin genome regions. All represents the open chromatin regions identified from glioma tissue samples. Overlap represents the open chromatin regions identified from both glioma tissues and CSF cfDNA. **(E)** The cfDNA fragment distribution in the open chromatin regions identified from CSF cfDNA in patients. **(F)** The top 50 transcription factors with the highest number of binding sites in each CSF cfDNA-identified open chromatin region. The bar plot at the top of the heatmap indicates total number of TFs in each genome region. The number of TF binding sites in each genome region is shown on the right side of the plot. The bar plot at the bottom shows the percentage of the top 50 TF binding sites within the total binding sites identified in genome regions. The red part indicates the ratio of the top 50 TF binding sites, and the green part indicates the binding sites contributed by other TFs. CSF, cerebrospinal fluid; cfDNA, cell-free DNA; TFs, transcription factors.

To investigate whether the diversity of the open chromatin state of glioma patients could be used as a marker in liquid biopsy, we further verified the chromatin accessibility features identified from tissues using CSF cfDNA. We identified a total of 72 genome regions of the tissue-derived peaks as open chromatin regions based on CSF cfDNA (p = 8.012416e−196) (**Figure 3A**). Compared with the surrounding genome regions, the open chromatin genome regions identified using cfDNA had higher read counts (**Figure 3B**). This indicates that the cfDNA located in these regions may be bound by proteins, such as transcription factors, which could provide a protective function. These cfDNA-derived open regions showed a clustered distribution throughout the whole genome. For example, there were several clusters of open chromatin regions on chromosomes 1, 16, and 21 (**Figure 3C**). Regions with a high density of open chromatin typically play critical roles in transcription regulation and provide clues to further investigate the gene regulation roles of these genome regions. To further explore the genomic features of cfDNA-derived open regions, we performed genome annotation (**Figure 3D**). The genomic features of these open chromatin regions identified using CSF cfDNA were distinct compared to those of tissue-derived regions. The percentage of peaks located in promoter regions was lower than that in tissue-derived peaks, while a higher number of peaks were related to introns compared to CSF cfDNA-derived peaks (**Figure 3D**). To investigate whether the open chromatin regions identified using cfDNA also exhibited high diversity, we examined the cfDNA fragment distribution in each genome region. Similar to the high diversity found in the tissue samples, the cfDNA fragment distribution in the open chromatin regions also showed high diversity (**Figure 3E**). In each cfDNA sample, some genome regions exhibited an extremely high count of cfDNA fragments (**Figure 3E**), indicating that these genome regions have high potential as biomarkers to classify different gliomas based on the cfDNA-derived open chromatin features. To further verify the reliability of the open chromatin regions identified using cfDNA, we searched the transcription factor binding site (TFBS) distribution states in these regions. The top 50 transcription factors (TFs) with the highest number of binding sites were identified, with Myc-associated zinc finger protein (MAZ) exhibiting the largest TFBS count (**Figure 3F**). MAZ is known to be closely related to glioma (32). Based on our analysis, we found that although these top 50 TFs account for a large portion of TFBSs in these open genome regions, especially in some regions, all TFBSs belonged to the top 50 TFs (**Figure 3F**). Some genome regions also contained a high variety of TFBSs, which could be bound by different TFs (**Supplementary Table 4**). Based on these results, we inferred that the cfDNA can not only be used to identify the open chromatin regions in glioma but also reveal the same high diversity observed in tissue samples.

### 3.4 Distinct open chromatin feature in LGG and GBM

**Figure 4:**
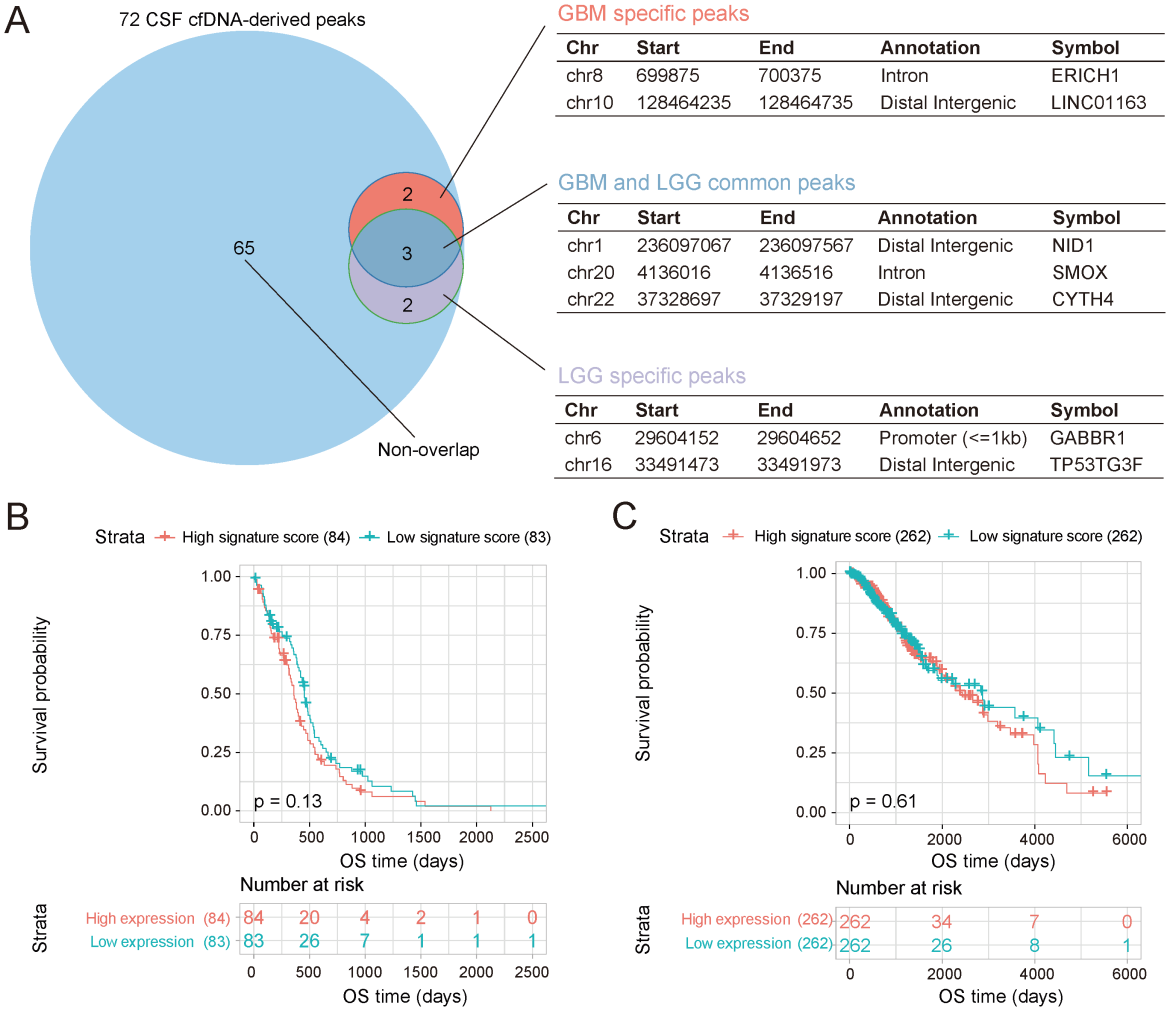
The specificity of CSF cfDNA-identified open chromatin regions. **(A)** Open chromatin regions overlapping with GBM and LGG accessible genome regions. Annotations of each chromatin region are shown. **(B)** Survival of GBM patients calculated based on GBM-specific open chromatin region-related genes. The colored lines represent the high- and low-expression levels of the patient groups, as indicated above the figure. **(C)** The survival of LGG patients calculated based on the LGG-specific open chromatin region-related genes. The colored lines represent the high- and low-expression levels of the patient groups, as indicated above the figure. CSF, cerebrospinal fluid; cfDNA, cell-free DNA; GBM, glioblastoma; LGG, low-grade glioma.

To further investigate whether CSF cfDNA-derived open chromatin states could reflect the differences among different subtypes of glioma, the overlapping regions were identified among 72 CSF cfDNA-derived and LGG- and GBM-specific open regions. Three open chromatin genome regions were found to overlap between LGG and GBM (**Figure 4A**). These results indicate that the genome positions of some open chromatin regions of LGG or GBM are very close and regulate the same genes, such as NID1, SMOX, and CYTH4 (**Figure 4A**). In addition to these common chromatin regions, subtype-specific genome regions were also found (**Figure 4A**), indicating the diversity of chromatin accessibility among different glioma subtypes. Chromatin accessible genome regions play important roles in gene expression regulation, and the open chromatin regions are usually closely related to super-enhancers. In order to investigate the gene expression regulation potential of these subtype-specific open chromatin regions, the super-enhancers were detected using the ROSE program based on two public ATAC-seq datasets. Among the open chromatin regions identified in both GBM and LGG, two genome regions derived from GBM and four genome regions derived from LGG were identified as super-enhancers (**Supplementary Figure 4A**). To further validate the reliability of these identified super-enhancers, the genomic features of these regions were shown in the UCSC Genome Browser. Hi-C signals, H3K27ac ChIP-seq peaks, and *cis*-Regulatory Element of ENCODE were all closely related to these open chromatin regions (**Supplementary Figure 4B**). In addition, the TF binding states of these open chromatin regions were investigated. TF binding sites were widely distributed in chromatin accessible genome regions (**Supplementary Figure 5**), and TFs could interact with multiple genome sites in these genome regions (**Supplementary Figure 6**), indicating that these open chromatin regions may play important roles in transcription regulation. Moreover, we found that p52 protein, which is an important NF-κB member, could bind with these genome regions (**Supplementary Figure 7**). This result also indicated the role of the open chromatin regions in transcription regulation. In addition, these open chromatin regions defined in our study could not only be used as novel biomarkers to classify the grade of glioma but also provide biological clues to investigate the development mechanisms of glioma. We also performed survival analysis using these glioma subtype-specific open genome region-related genes as gene signatures. Based on the gene signature, scores for each patient were calculated using ssGSEA, and then the relationship between the survival time and the gene set was identified. Although no significant differences were found between the high signature score and low signature score groups in either GBM (**Figure 4B**) or LGG (**Figure 4C**), the low score group exhibited slightly better survival states (**Figures 4B, C**). However, the expression levels of individual genes in the two datasets showed distinct phenomena. For GBM patients, no gene showed a significant association with survival time (**Supplementary Figure 8A**), which may be due to the poor survival rate of GBM. Meanwhile, for LGG patients, the expression levels of CYTH4, GABR1, and NID1 were all closely related to the survival time (p < 0.05) (**Supplementary Figure 8B**). Specifically, NID1 was related to open chromatin regions in both the LGG and GBM samples, demonstrating the different functions of genes between the two glioma subtypes and further confirming the diversity of gene transcription regulation between them.

### 3.5 Classification of glioma subtypes using open chromatin features identified from plasma cfDNA

**Figure 5:**
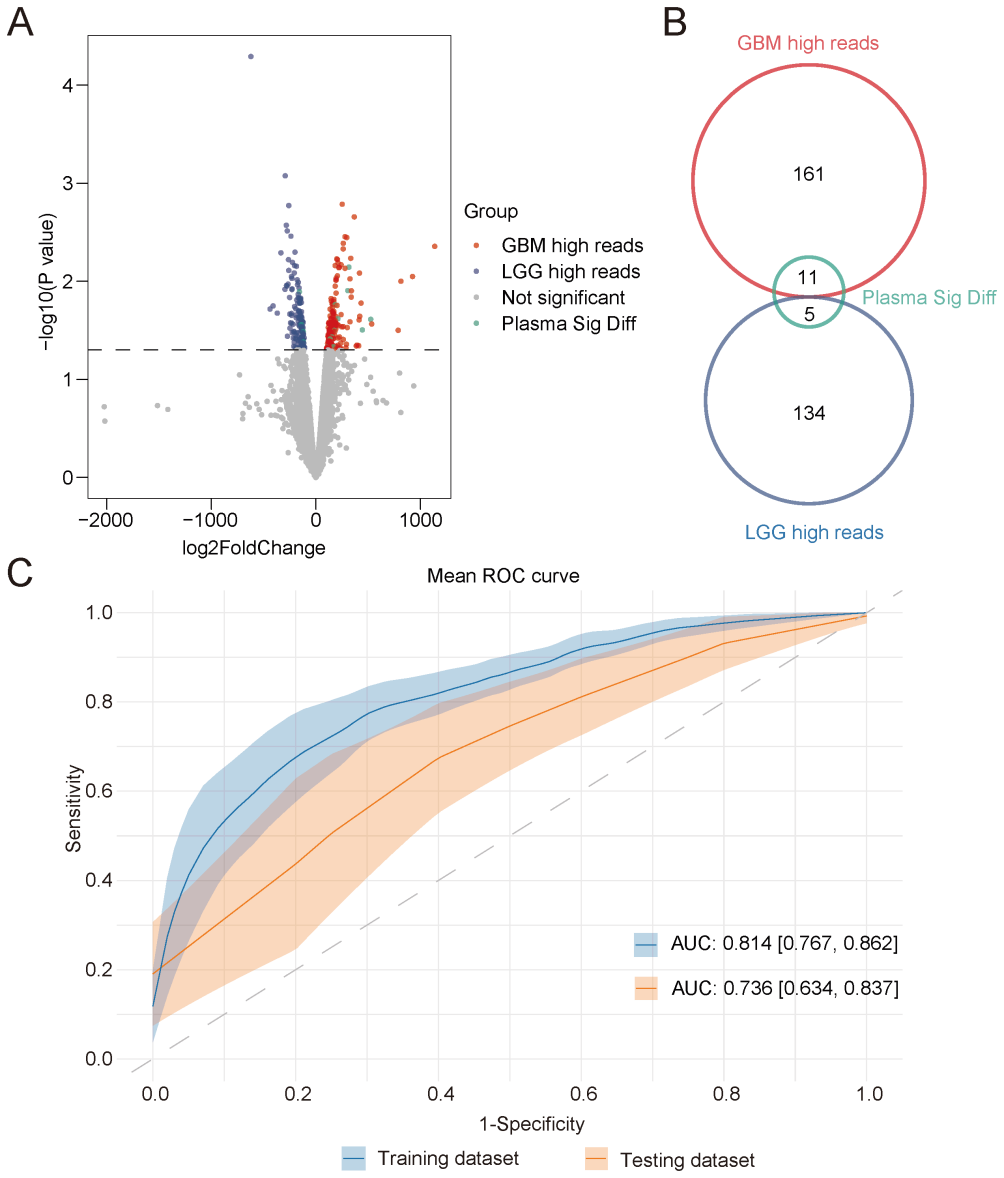
Classification of glioma patients based on open chromatin regions identified from cfDNA. **(A)** Significantly different open chromatin regions identified from GBM and LGG tissue samples. Significant differences in the genome regions identified using plasma cfDNA from glioma patients are also shown. GBM high reads represent the genome regions in GBM tissue samples with a high read number. LGG high reads represent the genome regions in LGG tissue samples with a high read number. Plasma Sig Diff represents genome regions showing significant differences in plasma cfDNA derived from GBM and LGG patients. Not significant represents genome regions with no significant difference between glioma tissue samples. **(B)** The number of genome regions of each group identified in **(A, C)** The ROC curve of the classifier constructed using plasma-derived significantly different genome regions in the training and testing datasets. **(D)** Unsupervised hierarchical clustering of 16 open chromatin markers selected for use in the prediction model in the training (left) and validation datasets (right). cfDNA, cell-free DNA; GBM, glioblastoma; LGG, low-grade glioma.

CSF collection is more acceptable than a brain tissue biopsy, as this procedure is still a minimally invasive treatment. To investigate whether the diverse open chromatin features could be identified using plasma cfDNA, which is more commonly used in liquid biopsy, we compared the distribution of cfDNA fragments among glioma subtype-specific accessible chromatin regions. We identified the significant differences in open chromatin regions between LGG and GBM, along with the genome regions exhibiting significantly different numbers of plasma cfDNA fragments (**Figure 5A**). To further investigate the biological function of the significantly differentially accessible chromatin regions, we performed functional analysis. As shown in **Supplementary Figure 9**, the glioma subtype-specific chromatin regions play diverse biological roles, indicating that these potential classification markers could also provide clues to demonstrate the glioma development mechanisms. We identified a total of 16 genome regions as significantly differentially accessible genome regions specific to glioma subtype using plasma cfDNA (**Figure 5B**). With these 16 genome regions, we constructed a glioma subtype classifier based on an XGBoost model with a public glioma plasma cfDNA 5hmC-sequencing dataset after a feature selection procedure. After 10-fold cross-validation, the AUC of the training dataset was 0.814 [0.767, 0.862], and the AUC of the testing dataset was 0.736 [0.634, 0.837] (**Figure 5C**). We also constructed a model with randomly selected genome regions, which showed lower AUC in both the training and testing datasets (**Supplementary Figure 10**). To verify that the classification of glioma subtypes was based on the open chromatin features derived from our study, we also compared the tumor fraction of each sample used in our construction dataset. As shown in **Supplementary Figure 11**, although the tumor fraction of each sample was diverse, there was no significant difference between the two glioma subtypes. This result demonstrated that the classified glioma subtype was independent of the concentration of cfDNA caused by the different grades of glioma. Based on these results, we conclude that a portion of the chromatin accessibility features of glioma subtypes can be detected using plasma cfDNA, and these features may also provide clues for identifying glioma subtypes using plasma cfDNA.

## 4 Discussion

Similar to most common primary central nervous system malignant tumors in adults, gliomas are highly heterogeneous at the histopathological and molecular levels, which are linked to significant variability in clinical outcomes (33, 34). Accurate and robust classification of the patient is crucial for personalized care and treatment of glioma (35). In this study, we investigated the chromatin accessibility features of different subtypes of gliomas based on both CSF and plasma cfDNA. Different from classical biomarkers, using the chromatin accessibility state as a liquid biopsy marker presents multiple advantages in capturing heterogeneity at the gene transcription regulation level and can also be used to further explore the mechanisms underlying glioma origin and development.

The open chromatin regions that we detected in our study were identified as 501-bp genome regions. We consider these types of liquid biopsy markers to have an anti-degradation ability, which is an important feature, especially in blood circulation systems. cfDNA can be cleared from blood in approximately 16 min to 2.5 h (36, 37). Due to its short half-life, several features contained in cfDNA face a risk of degradation due to the DNA nuclease in the fluid circulation system and other clearance mechanisms (38). Driver gene mutations are the most widely used cfDNA markers for different types of cancer, including glioma (13). The detection of cancer-related mutations is usually performed using a target capture method, which is greatly influenced by the existence of mutations contained in cfDNA fragments. The sensitivity of gene mutation detection is closely related to the degree of cfDNA degradation. With a single base target, driver gene mutations may be lost due to the elimination of mutations containing cfDNA, leading to false-negative results. More importantly, tumor-derived cfDNA is limited in glioma patients, even in CSF, which directly interacts with brain tissues (13). This phenomenon is another obstacle to driver mutation-based liquid biopsy for glioma. The 501-bp genome regions used in our detection were larger than the typical length of cfDNA and may have included a number of cfDNA fragments (**Supplementary Figure 12**). Chromatin accessibility can be reflected by the number of fragments of cfDNA, which is also an index of cfDNA degradation. The cfDNA fragment count within a target genome region is closely related to the chromatin accessibility and the TF binding states. Accessible chromatin can be degraded by DNA nuclease, whereas the binding of TFs can protect the genomic DNA from degrading. Furthermore, due to the complexity and heterogeneity of cancer and the expanding repertoire of research tools and refined model systems, cancer is treated as a systemic disease (39). This definition of cancer provides an opportunity to detect chromatin accessibility changes in genomes derived from different systems, in addition to the specifically targeted tumor tissues, to comprehensively illustrate the characteristics of cancers. That meant that the open chromatin states identified from cfDNA in our study have the potential to reflect epigenetic changes not only in tumor tissues but also in other systems, such as the immune system. Cancer cells can secrete and release tumor-derived nucleic acids, which can influence the immune cells (40). In addition, cfDNA fragments could be used to infer nucleosome position, which was closely related to chromatin accessibility. This cfDNA-derived information could be used to infer the tissue origin of cfDNA based on the cell type-specific chromatin accessibility patterns and demonstrate the change of the immune system based on the epigenetic features. These open chromatin features could provide a new way to overcome the obstacle of the BBB in glioma liquid biopsy and may also provide a chance to trace the changes of immune response during the disease and treatment procedure. Compared with established molecular markers used in glioma molecular subtyping, such as IDH mutation, 1p/19q deletion, and MGMT methylation status, the open chromatin feature-based methods could capture the characteristics of glioma in a dynamic way. Epigenetic disorder is an important feature of cancer, and the change of epigenetic feature is closely related with the development of cancer, including glioma. The accessibility of chromatin as an epigenetic feature plays key roles in disease process. Based on this reason, the open chromatin feature could not only classify the subtype of glioma but also, with great potential, track the development of glioma, especially during the therapy procedure.

The distribution of cfDNA fragments in our selected genome regions was closely related to the nucleosome positioning and the binding states of TFs. cfDNA derived from genome regions lacking nucleosomes can be easily digested by nuclease, while TF-bound genomic DNA can be preserved, appearing in high-throughput sequencing data. These two events are essential components of gene transcription regulation, and abnormalities in either of these events are important features of cancer (25, 41). Based on these results, the open chromatin state identified from cfDNA in our study can not only be used to identify glioma patients but also shed light on investigating glioma-related gene transcription regulation. The gene expression in gliomas shows high diversity and could be used to classify GBM into different transcriptional subtypes (42). Gene expression is regulated by TFs that bind to open chromatin regions (43). The open chromatin states identified in our study also exhibited high diversity among patients (**Figure 3E**). The predicted TF binding sites contained in these genome regions were also distinct among the samples (**Figure 3F**). Based on these results, we conclude that activated gene transcription regulation pathways vary among different glioma patients. This means that our results also provide a novel method to investigate the formation mechanisms of heterogeneous gliomas based on cfDNA, instead of using tumor tissues.

The chromatin accessibility was closely related to gene expression. Based on this, we inferred that the expression level of subtype-specific open chromatin regions related genes may also show subtype characteristics. Although an investigation was performed using TCGA gene expression datasets, no significant relevance was found between these genes and the survival time. This may be caused by the non-linear relationship between chromatin accessibility and gene expression (44); other DNA epigenetic features, such as DNA methylation, which could influence the expression of genes, should also be considered.

Due to the rapid development of NGS, high-throughput sequencing-based detection methods can reveal much more information about the cfDNA distribution. However, low-throughput cfDNA detection methods are still more convenient compared with NGS. For this reason, GBM- and LGG-specific genome regions with differential chromatin accessibility were also amplified in CSF derived from several types of patients, including post-operative glioma patients, hydrocephalus patients, intracranial infection patients, meningioma patients, and pituitary adenoma patients. However, no sample type specificity was detected (**Supplementary Figure 13**). We infer that these genome regions may play roles in the transcription regulation of inflammation- and immunity-related genes, as the annotated genes are closely related to inflammation and immune response (45–49). This relationship was also validated using a ChIP-seq dataset, which was performed using the TWEAK-treated U-87 MG cell line. The binding states of the p52 protein were investigated. The p52 protein binding signals could be found in all open chromatin regions identified in our study, indicating that the genes related to open chromatin regions played roles in NF-κB-linked inflammation (**Supplementary Figure 7**). All types of samples used in our target amplification experiment presented inflammation or infection, which may be the reason for our amplification results. This represents a limitation of our study, and further validation should be performed to explain these effects.

In addition, the major limitation of our study is the relatively small size of the cohorts studied. In our study, a total of four patients diagnosed with LGG and GBM were enrolled. The high inter- and intra-tumor heterogeneity of gliomas was demonstrated, especially with the development of single-cell sequencing techniques in both gene expression and open chromatin state levels (50, 51). In addition, the tumor microenvironment of glioma also dramatically changed during the evolution of the tumor (52). Although the open chromatin features identified in our study were verified using several high-throughput sequencing methods performed using tumor and non-tumor organoid or cell lines, such as ATAC-seq performed using organoids and ChIP-seq performed using cell lines, examining a larger cohort may increase the specificity of the test. A cohort including more patients with different baseline information and progression status is warranted to confirm the findings on the open chromatin features and the subtype of glioma, and to validate the reliability of these accessible chromatin features. Moreover, the clinical relevance of our subtype-specific chromatin accessibility features may be increased if the cohort, including open chromatin state information, could be used in further study. Furthermore, combining the gene expression information derived from paired samples could further demonstrate the biological function of the chromatin regions defined in our study.

## 5 Conclusions

In this study, cfDNA in both CSF and plasma from patients with glioma was successfully analyzed and compared with the chromatin accessibility features identified from paired brain tumor tissues. Based on the cfDNA distribution features in open chromatin regions, the patient-specific chromatin accessibility was determined. The high diversity of open chromatin states among the glioma patients and glioma subtypes was also reflected using cfDNA, indicating their potential as liquid biopsy markers for glioma. Furthermore, the diversity of TF binding states was investigated by analyzing cfDNA-identified activated transcription regulation elements. In conclusion, this study provides a new type of epigenetic biomarker for the detection of glioma, which may overcome the obstacles caused by the BBB in glioma liquid biopsy and expand the use of cfDNA to investigate the complex mechanisms of gliomas.

## Data Availability

The raw sequence data reported in this paper have been deposited in the Genome Sequence Archive (Genomics, Proteomics & Bioinformatics 2025) in National Genomics Data Center (Nucleic Acids Res 2025), China National Center for Bioinformation / Beijing Institute of Genomics, Chinese Academy of Sciences (GSA-Human: HRA015163) that are publicly accessible at https://ngdc.cncb.ac.cn/.

## Supplementary material

Supplementary Figure 1 The fragment length distribution of sequencing libraries. **(A)** The fragment length distribution of SALP-seq libraries derived tissue samples from different patient. **(B)** The fragments length of plasma derived cfDNA sequencing libraries. **(C)** The fragment length of cfDNA sequencing libraries constructed using CSF cfDNA samples.

Supplementary Figure 2 The diversity of open chromatin regions identified from different tissue samples. **(A)** The relationship between open chromatin region diversity and common peaks. The coefficient of variation of the peak numbers derived from different patients in 1 MB genome windows were calculated, and are shown as a heatmap. The density of common peaks among glioma patients was identified using a 1 MB genome window, and illustrated using a line plot. **(B)** The coefficient of variation (CV) of open chromatin regions throughout the whole genome. The CVs were calculated for each 1 MB genome window. **(C)** The genomic features of open chromatin regions identified in each glioma patient.

Supplementary Figure 3 The validation of open chromatin regions. **(A)** The reads distribution in identified 12066 open chromatin regions of 15 patient-derived GBM cell lines and a normal human astrocytes cell line. The cell lines marked with red color were GBM cell lines and cell lines marked with green was astrocytes cell line. **(B)** The reads distribution in identified 12066 open chromatin regions of 10 GBM human cortical organoids and paired margin samples. Tumor: GBM organoid samples. Margin: paired margin samples. **(C)** The reads distribution of open chromatin regions with significant differential chromatin accessibility between GBM tumor organoids and margin samples. **(D)** The fold change and p value of open chromatin regions with significant differential chromatin accessibility among organoid and margin samples. The number of peaks under each condition were showed.

Supplementary Figure 4 The super-enhancers identified in subtype-specific open chromatin regions. **(A)** The super-enhancers identified in GBM and LGG related open chromatin regions. Two ATAC-seq data sets derived from GBM (SRR33281919) and LGG (SRR33281847) contained in GSE295378 were used to investigate the relationship between the open chromatin regions and super-enhancer. The raw sra data was transferred to fastq using fastq-dump program with –split-3 parament. And the reads were aligned to hg38 reference genome using Bowtie2, then ROSE program was used to identified the super-enhancer. **(B)** The genome feature of open chromatin regions. The genome features open chromatin regions identified as super-enhancers were showed in UCSC genome browser. The Hi-C track, CRE (cis-Regulatory Element) and H3K27ac ChIP-seq peak track were showed to validation the reliability of the identified super-enhancer. Open chromatin regions identified in this study were highlighted.

Supplementary Figure 5 The distribution of transcription factor binding sites in open chromatin regions. The open chromatin regions were highlighted and H3K27ac marker track, ReMap density tranck, ReMap ChIP-seq track were also showed to validate the chromatin accessibility of identified genome regions. The transcription factor binding sites were predicted in each highlighted genome regions, and the number of binding sites covered each base of genome regions were calculated and showed in zoomed in barplot. **(A)** Open chromatin region related to NID1; **(B)** Open chromatin region related to SMOX; **(C)** Open chromatin region related to CYTH4.

Supplementary Figure 6 The type of transcription factors with binding sites in open chromatin regions. The number of binding sites were count for each transcription factor with binding sites in open chromatin regions. The transcription factor which were with more than 5 binding sites in genome regions were showed.

Supplementary Figure 7 The binding state of p52 protein in open chromatin regions. **(A)** The binding state of p52 protein in TWEAK treated and untreated U-87 MG cell line. The highlighted genome regions were open chromatin regions identified by SALP-seq. TW p52: TWEAK treated U-87 MG ChIP-seq performed using p52 antibody; UT p52: untreated U-87 MG ChIP-seq performed using p52 antibody. **(B)** Reads distribution of open chromatin regions. Open chromatin regions were divided with 100 bp window, read counts in each window were calculated for each sample. UT p52 rep 1: untreated U-87 MG ChIP-seq performed using p52 antibody replicate 1; UT p52 rep2: untreated U-87 MG ChIP-seq performed using p52 antibody replicate 2; TW p52 rep1: TWEAK treated U-87 MG ChIP-seq performed using p52 antibody replicate 1; TW p52 rep2: TWEAK treated U-87 MG ChIP-seq performed using p52 antibody replicate 2.

Supplementary Figure 8 Survival curve derived from open chromatin region-related genes. **(A)** The survival rate of GBM patients calculated using GBM-specific open genome region-related genes. **(B)** The survival rate of GBM patients calculated using LGG-specific open genome region-related genes. The colored lines represent the high and low expression levels of specific genes in each patient group, as indicated above the figure.

Supplementary Figure 9 Pathway enrichment analysis. **(A)** KEGG enrichment analysis of the subtype-specific accessible chromatin regions. **(B)** GO enrichment analysis of the subtype-specific accessible chromatin regions.

Supplementary Figure 10 The mean ROC curve of classifier constructed with random selected genome regions.

Supplementary Figure 11 The comparison of tumor fraction between different glioma subtype. **(A)** The distribution of tumor fraction in LGG and GBM samples. **(B)** The comparison of tumor fraction between LGG and GBM. **(C)** The genome feature of samples with lowest and highest tumor fraction in both of LGG and GBM.

Supplementary Figure 12 The comparison of reads distribution between open chromatin regions and mutation related genome regions. The read counts of cfDNA derived from CSF and plasma from 4 patients were calculate in open chromatin regions and genome regions containing well known cancer related mutation collected from MSK-IMPACT gene panel. **(A)** Reads distribution in CSF cfDNA. **(B)** Reads distribution in CSF cfDNA. ***: Wilcoxon test p < 0.001.

Supplementary Figure 13 The amplification of glioma subtype-specific accessible chromatin regions. A total of 7 target regions located in the genome regions listed in **Figure 4A** were amplified using CSF cfDNA samples derived from post-operation glioma patients, non-glioma tumor patients, and non-tumor patients. Genomic DNA extracted from the peripheral blood of a healthy donor was used as a positive control. The amplification primers used for each genome region are listed in **Supplementary Table 5**. Glioma post-operation 1: Grade 4 glioma patient with post-operative intracranial infection. Glioma post-operation 2: Grade 1 glioma patient with post-operative intracranial infection. Glioma post-operation 3: Grade 4 glioma patient with post-operative intracranial infection. Glioma post-operation 4: Grade 4 glioma patient with post-operative intracranial infection. Glioma post-operation 5: Grade 4 glioma patient with post-operative intracranial infection.

Supplementary Table 1 Clinical information of donors enrolled in this study.

Supplementary Table 2 The barcode adaptor sequence in Tn5-SALP-seq.

Supplementary Table 3 The mappable ration and reads number.

Supplementary Table 4 The transcription factor binding site number in CSF identified chromatin open regions.

Supplementary Table 5 The sequence of primers used for glioma subtypes specific genome regions amplification.
