# Supplementary figures and images for: Chromatin accessibility derived from cfDNA serves as a novel classification biomarker of glioma
Source: Front Oncol. 2025 Dec 15;15:1688625. doi: 10.3389/fonc.2025.1688625 (PMC12745158; doi:10.3389/fonc.2025.1688625)

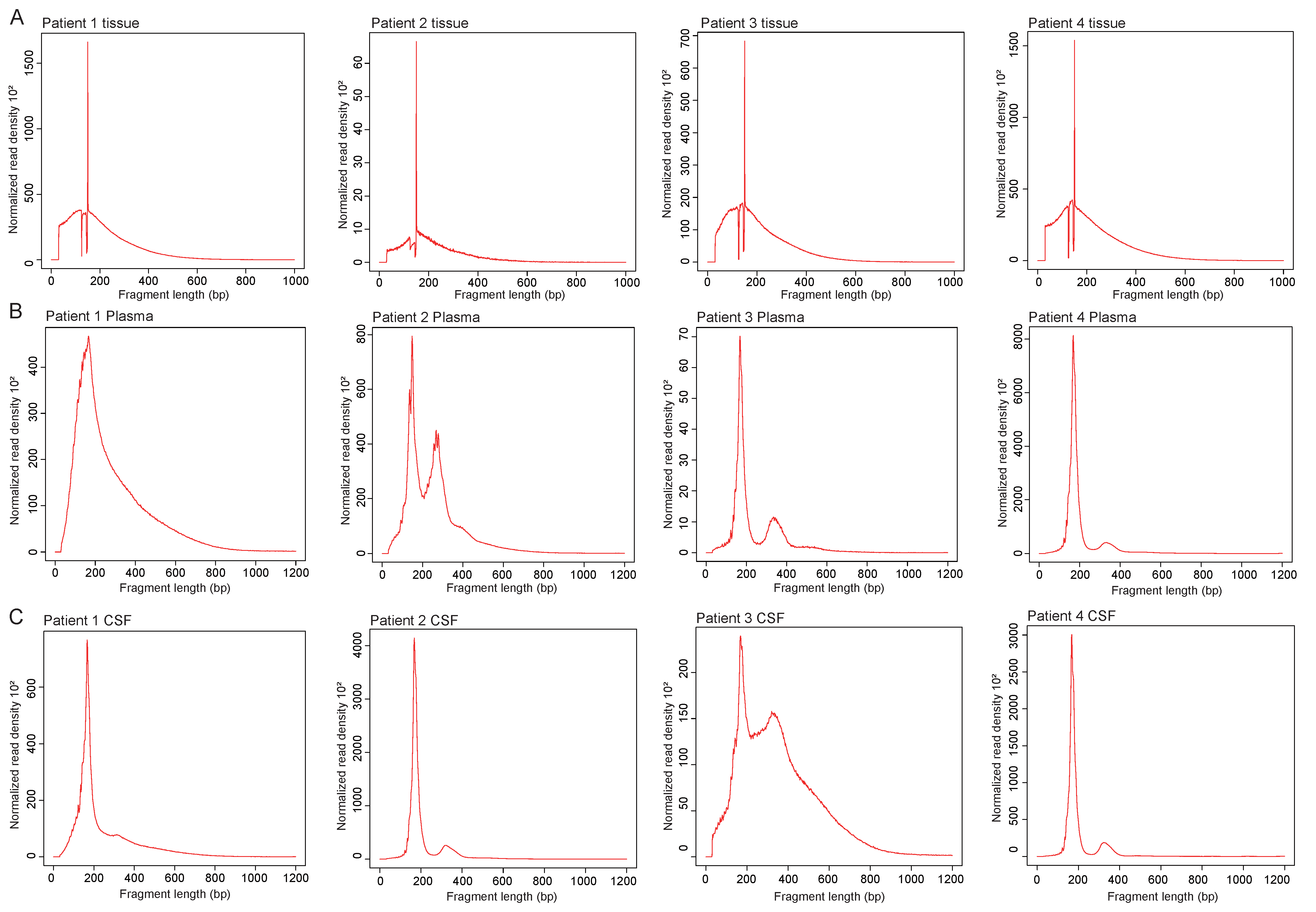

Supplement: Supplementary Figure 1 — The fragment length distribution of sequencing libraries. (A) The fragment length distribution of SALP-seq libraries derived tissue samples from different patient. (B) The fragments length of plasma derived cfDNA sequencing libraries. (C) The fragment length of cfDNA sequencing libraries constructed using CSF cfDNA samples. [file Image1.tiff]

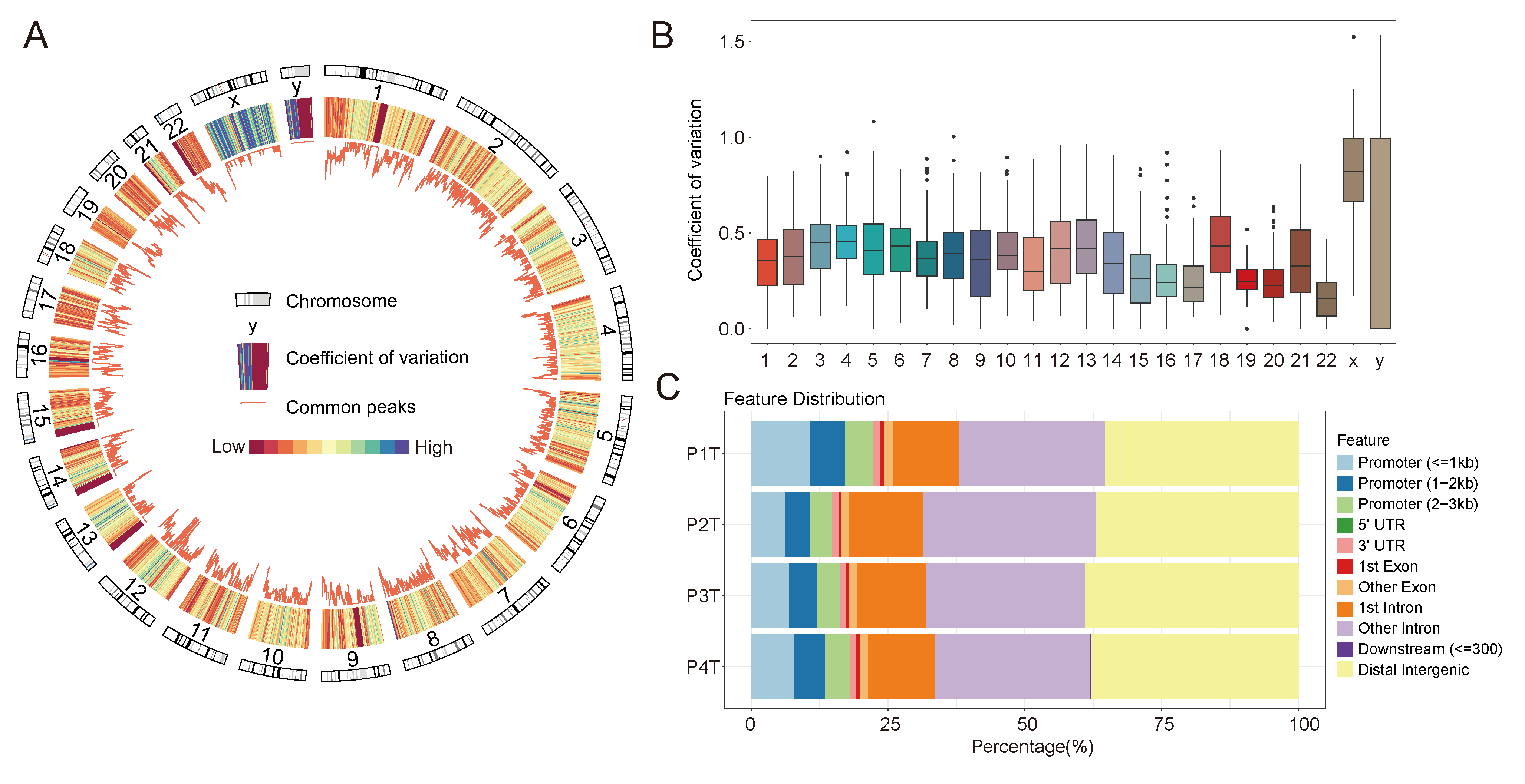

Supplement: Supplementary Figure 2 — The diversity of open chromatin regions identified from different tissue samples. (A) The relationship between open chromatin region diversity and common peaks. The coefficient of variation of the peak numbers derived from different patients in 1 MB genome windows were calculated, and are shown as a heatmap. The density of common peaks among glioma patients was identified using a 1 MB genome window, and illustrated using a line plot. (B) The coefficient of variation (CV) of open chromatin regions throughout the whole genome. The CVs were calculated for each 1 MB genome window. (C) The genomic features of open chromatin regions identified in each glioma patient. [file Image2.tif]

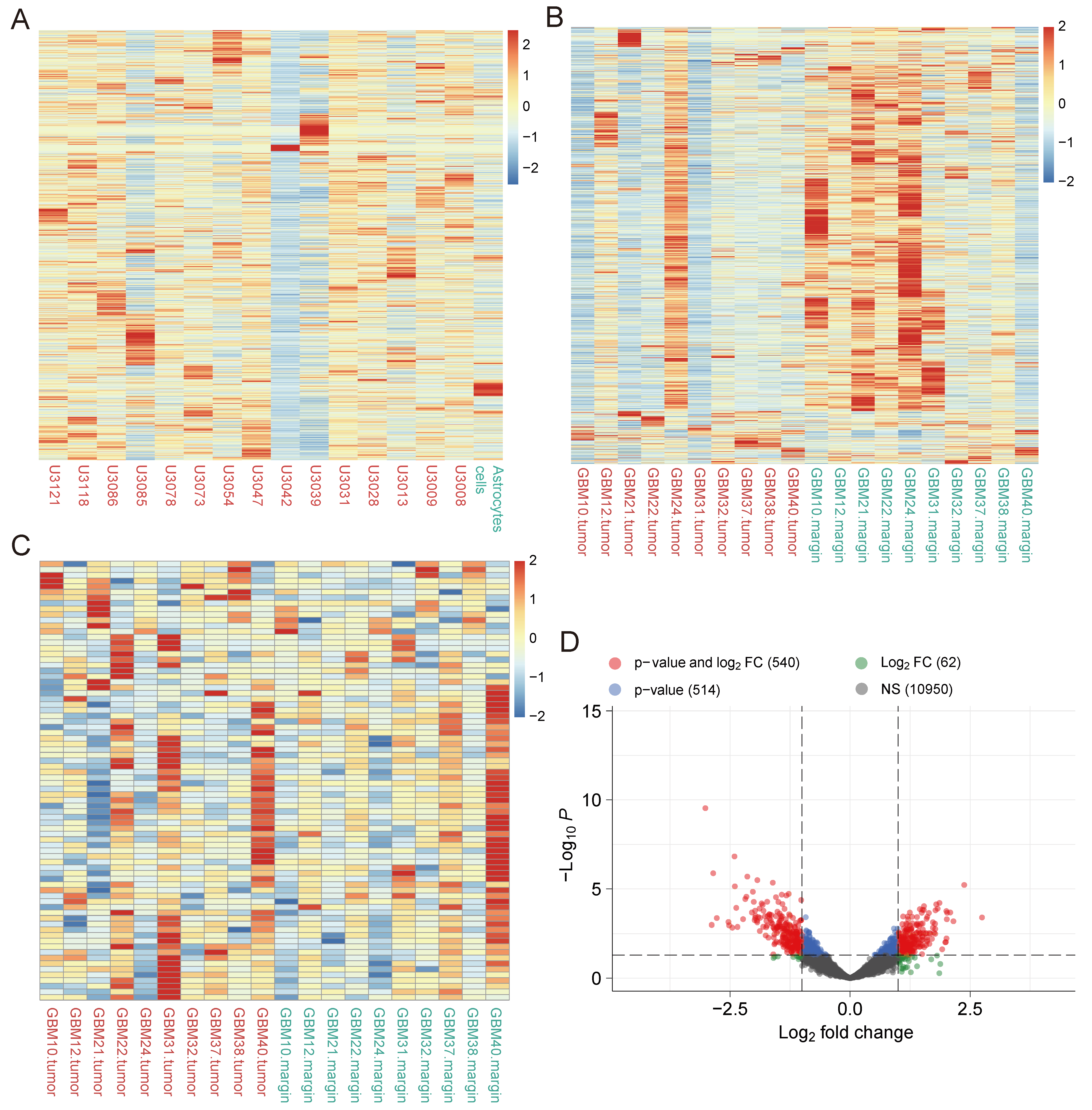

Supplement: Supplementary Figure 3 — The validation of open chromatin regions. (A) The reads distribution in identified 12066 open chromatin regions of 15 patient-derived GBM cell lines and a normal human astrocytes cell line. The cell lines marked with red color were GBM cell lines and cell lines marked with green was astrocytes cell line. (B) The reads distribution in identified 12066 open chromatin regions of 10 GBM human cortical organoids and paired margin samples. Tumor: GBM organoid samples. Margin: paired margin samples. (C) The reads distribution of open chromatin regions with significant differential chromatin accessibility between GBM tumor organoids and margin samples. (D) The fold change and p value of open chromatin regions with significant differential chromatin accessibility among organoid and margin samples. The number of peaks under each condition were showed. [file Image3.tiff]

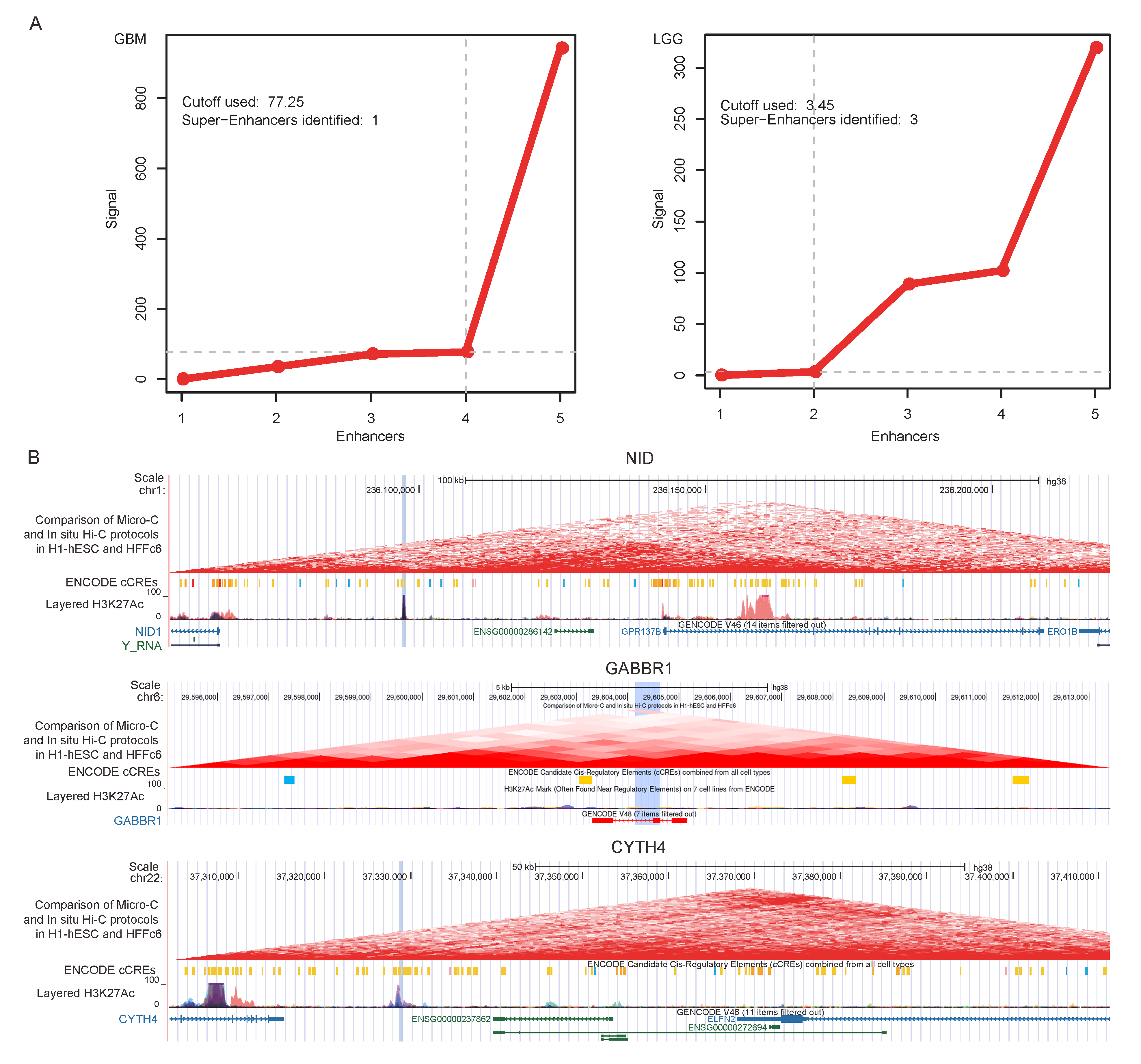

Supplement: Supplementary Figure 4 — The super-enhancers identified in subtype-specific open chromatin regions. (A) The super-enhancers identified in GBM and LGG related open chromatin regions. Two ATAC-seq data sets derived from GBM (SRR33281919) and LGG (SRR33281847) contained in GSE295378 were used to investigate the relationship between the open chromatin regions and super-enhancer. The raw sra data was transferred to fastq using fastq-dump program with –split-3 parament. And the reads were aligned to hg38 reference genome using Bowtie2, then ROSE program was used to identified the super-enhancer. (B) The genome feature of open chromatin regions. The genome features open chromatin regions identified as super-enhancers were showed in UCSC genome browser. The Hi-C track, CRE (cis-Regulatory Element) and H3K27ac ChIP-seq peak track were showed to validation the reliability of the identified super-enhancer. Open chromatin regions identified in this study were highlighted. [file Image4.tiff]

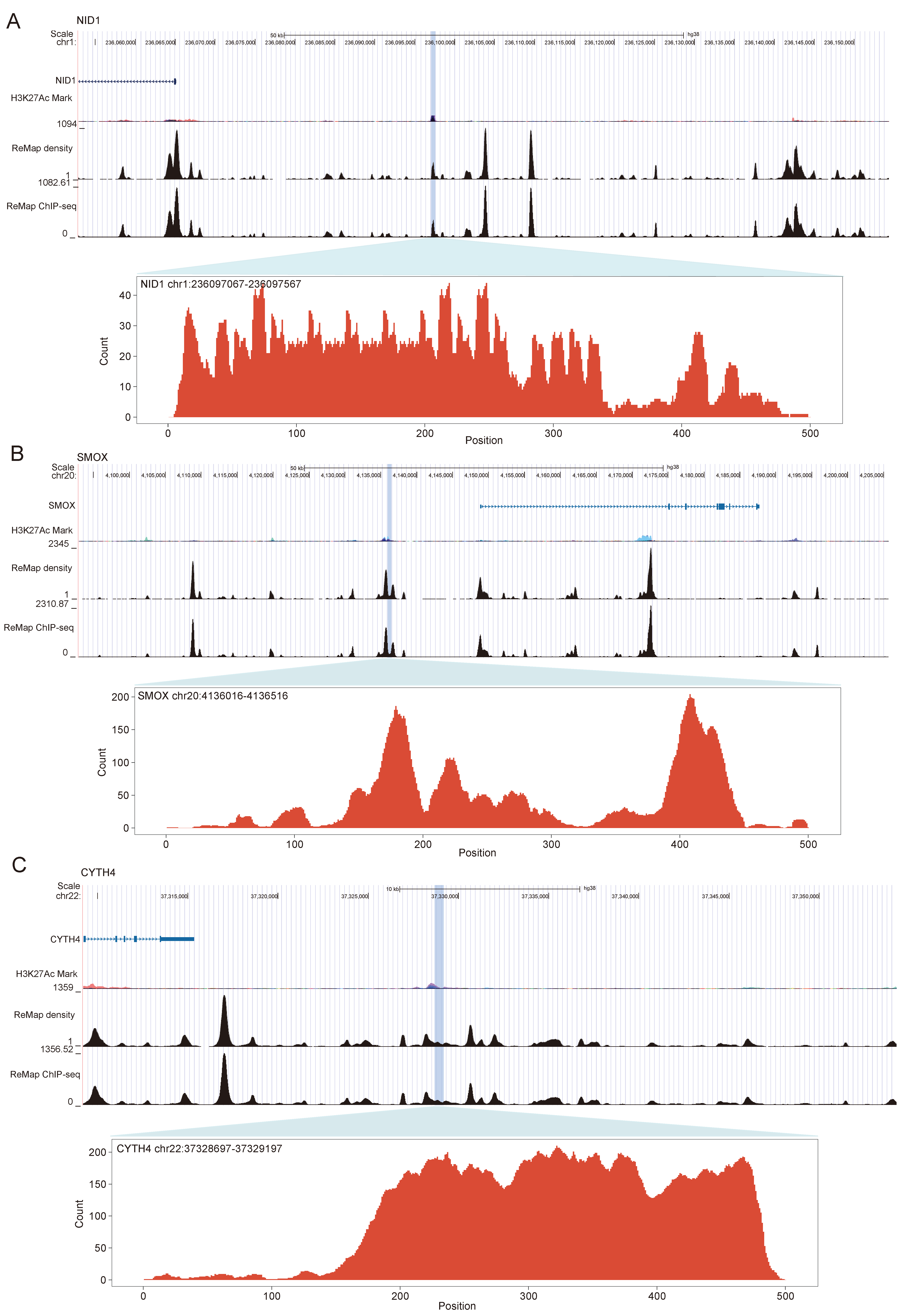

Supplement: Supplementary Figure 5 — The distribution of transcription factor binding sites in open chromatin regions. The open chromatin regions were highlighted and H3K27ac marker track, ReMap density tranck, ReMap ChIP-seq track were also showed to validate the chromatin accessibility of identified genome regions. The transcription factor binding sites were predicted in each highlighted genome regions, and the number of binding sites covered each base of genome regions were calculated and showed in zoomed in barplot. (A) Open chromatin region related to NID1; (B) Open chromatin region related to SMOX; (C) Open chromatin region related to CYTH4. [file Image5.tif]

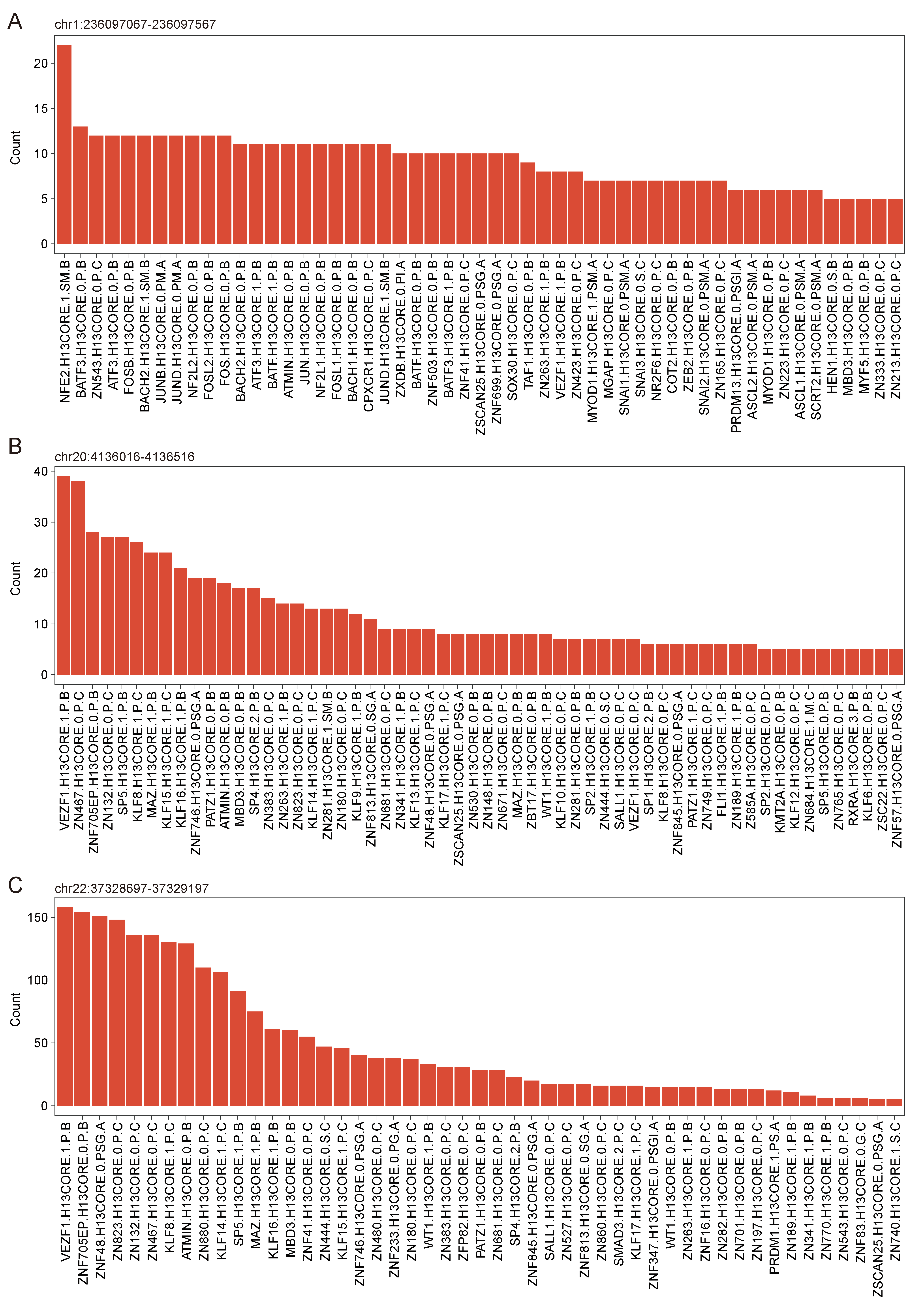

Supplement: Supplementary Figure 6 — The type of transcription factors with binding sites in open chromatin regions. The number of binding sites were count for each transcription factor with binding sites in open chromatin regions. The transcription factor which were with more than 5 binding sites in genome regions were showed. [file Image6.tif]

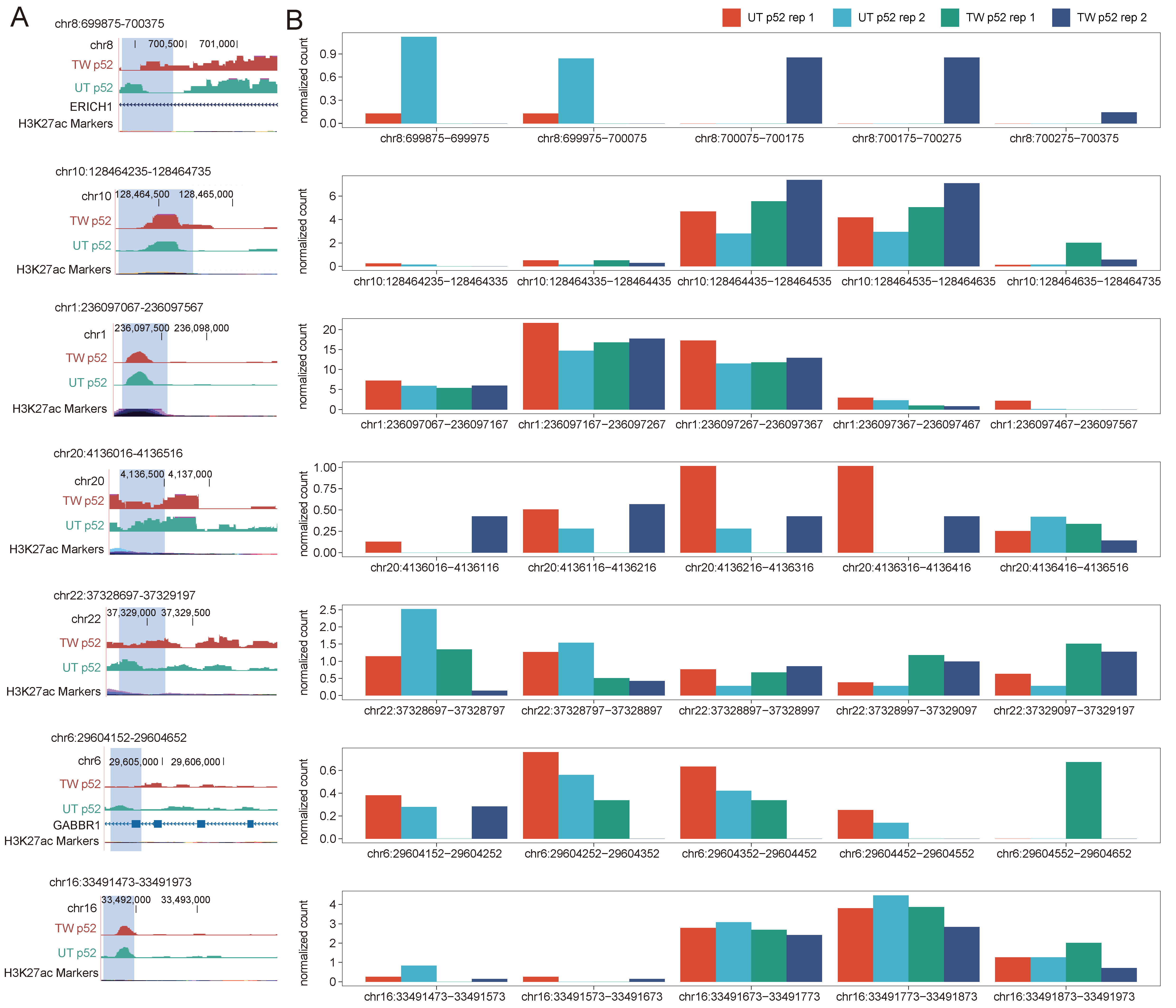

Supplement: Supplementary Figure 7 — The binding state of p52 protein in open chromatin regions. (A) The binding state of p52 protein in TWEAK treated and untreated U-87 MG cell line. The highlighted genome regions were open chromatin regions identified by SALP-seq. TW p52: TWEAK treated U-87 MG ChIP-seq performed using p52 antibody; UT p52: untreated U-87 MG ChIP-seq performed using p52 antibody. (B) Reads distribution of open chromatin regions. Open chromatin regions were divided with 100 bp window, read counts in each window were calculated for each sample. UT p52 rep 1: untreated U-87 MG ChIP-seq performed using p52 antibody replicate 1; UT p52 rep2: untreated U-87 MG ChIP-seq performed using p52 antibody replicate 2; TW p52 rep1: TWEAK treated U-87 MG ChIP-seq performed using p52 antibody replicate 1; TW p52 rep2: TWEAK treated U-87 MG ChIP-seq performed using p52 antibody replicate 2. [file Image7.tif]

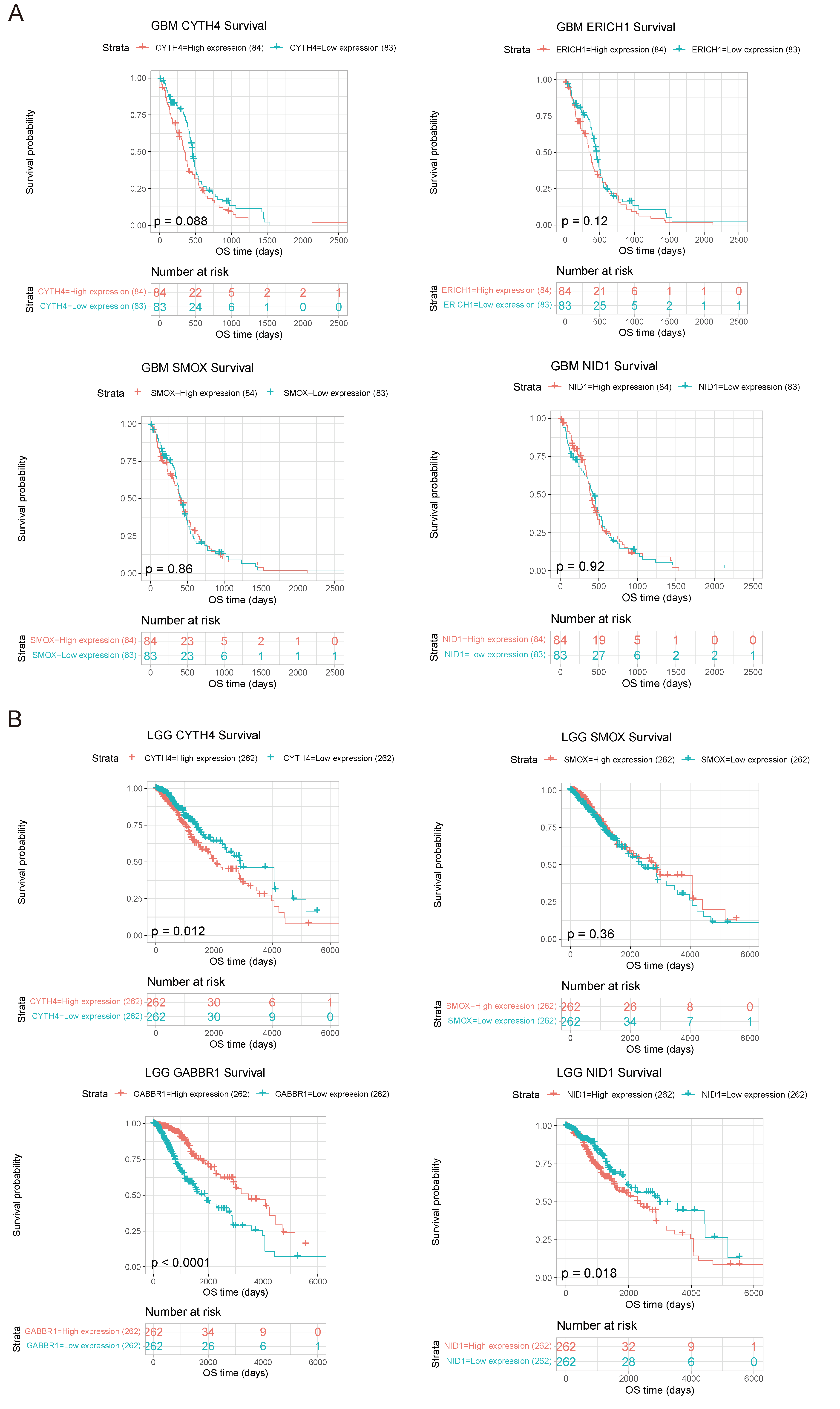

Supplement: Supplementary Figure 8 — Survival curve derived from open chromatin region-related genes. (A) The survival rate of GBM patients calculated using GBM-specific open genome region-related genes. (B) The survival rate of GBM patients calculated using LGG-specific open genome region-related genes. The colored lines represent the high and low expression levels of specific genes in each patient group, as indicated above the figure. [file Image8.tif]

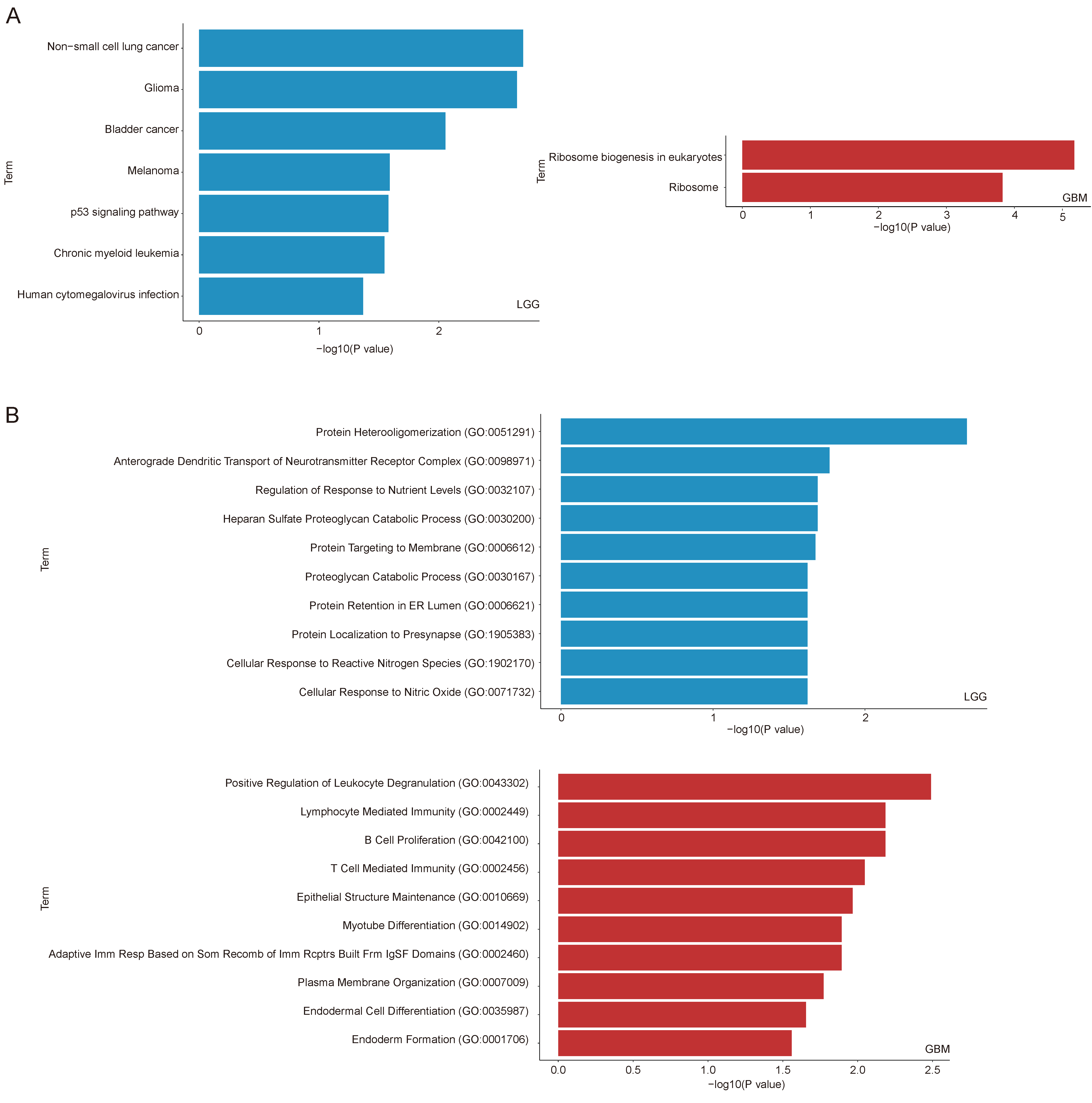

Supplement: Supplementary Figure 9 — Pathway enrichment analysis. (A) KEGG enrichment analysis of the subtype-specific accessible chromatin regions. (B) GO enrichment analysis of the subtype-specific accessible chromatin regions. [file Image9.tiff]

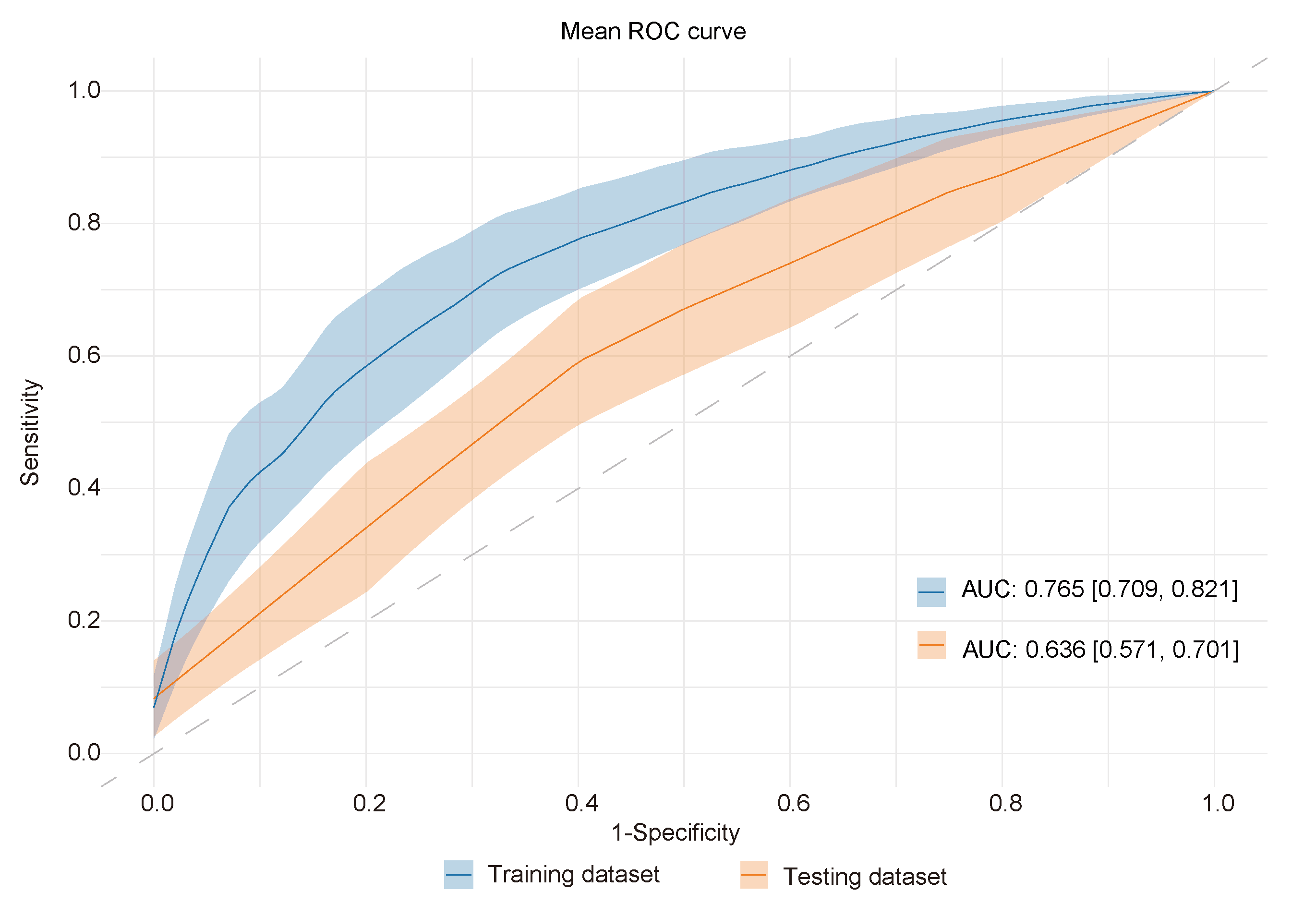

Supplement: Supplementary Figure 10 — The mean ROC curve of classifier constructed with random selected genome regions. [file Image10.tif]

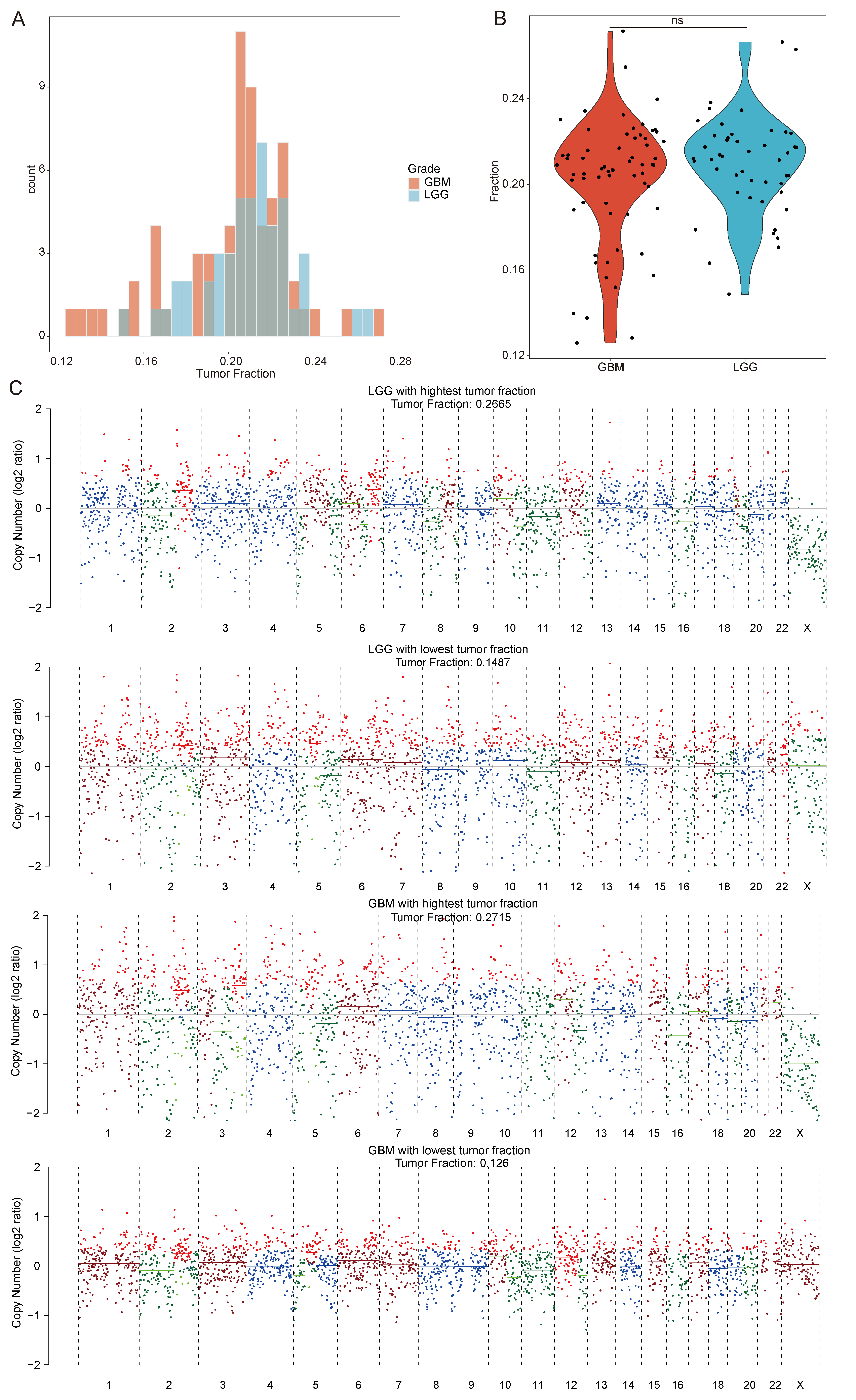

Supplement: Supplementary Figure 11 — The comparison of tumor fraction between different glioma subtype. (A) The distribution of tumor fraction in LGG and GBM samples. (B) The comparison of tumor fraction between LGG and GBM. (C) The genome feature of samples with lowest and highest tumor fraction in both of LGG and GBM. [file Image11.tiff]

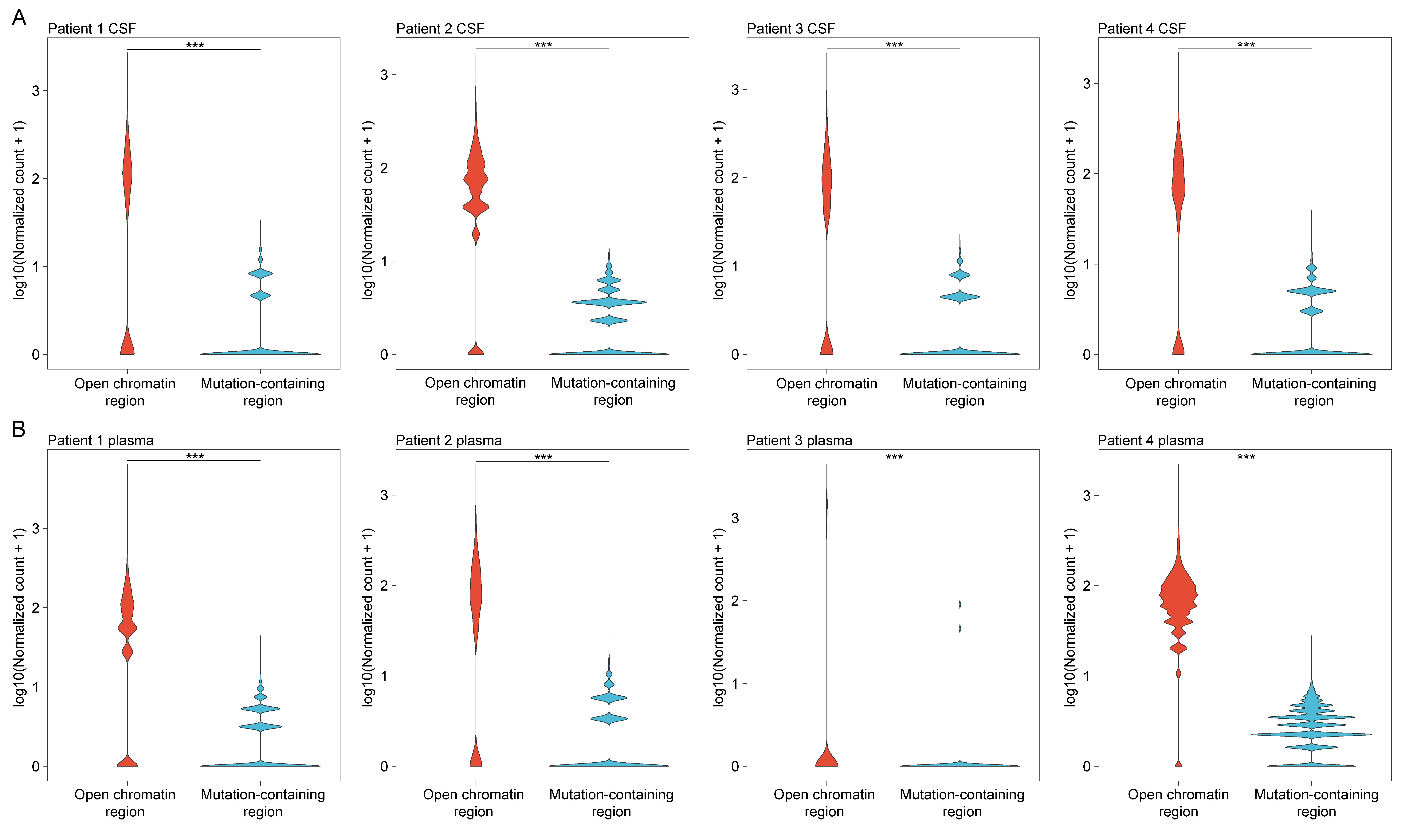

Supplement: Supplementary Figure 12 — The comparison of reads distribution between open chromatin regions and mutation related genome regions. The read counts of cfDNA derived from CSF and plasma from 4 patients were calculate in open chromatin regions and genome regions containing well known cancer related mutation collected from MSK-IMPACT gene panel. (A) Reads distribution in CSF cfDNA. (B) Reads distribution in CSF cfDNA. ***: Wilcoxon test p < 0.001. [file Image12.tiff]

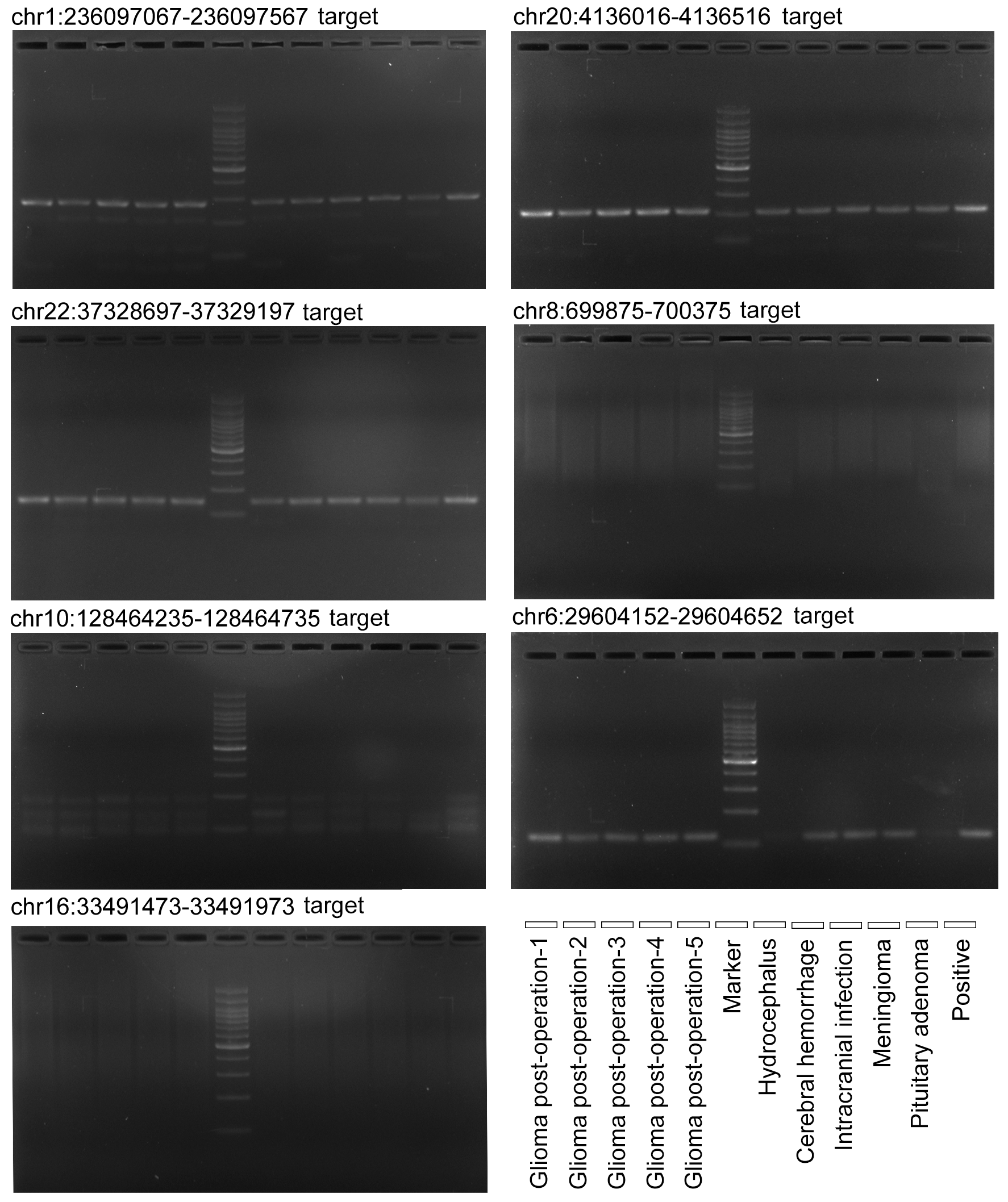

Supplement: Supplementary Figure 13 — The amplification of glioma subtype-specific accessible chromatin regions. A total of 7 target regions located in the genome regions listed in Figure 4A were amplified using CSF cfDNA samples derived from post-operation glioma patients, non-glioma tumor patients, and non-tumor patients. Genomic DNA extracted from the peripheral blood of a healthy donor was used as a positive control. The amplification primers used for each genome region are listed in Supplementary Table 5. Glioma post-operation 1: Grade 4 glioma patient with post-operative intracranial infection. Glioma post-operation 2: Grade 1 glioma patient with post-operative intracranial infection. Glioma post-operation 3: Grade 4 glioma patient with post-operative intracranial infection. Glioma post-operation 4: Grade 4 glioma patient with post-operative intracranial infection. Glioma post-operation 5: Grade 4 glioma patient with post-operative intracranial infection. [file Image13.tif]
