# Supplementary material for: Chromatin accessibility derived from cfDNA serves as a novel classification biomarker of glioma
Source: Front Oncol. 2025 Dec 15;15:1688625. doi: 10.3389/fonc.2025.1688625 (PMC12745158; doi:10.3389/fonc.2025.1688625)
Supplement: Supplementary Table 1 — Clinical information of donors enrolled in this study. [file Table1.docx]

| **Patient ID** | **Gender** | **Age** | **Diagnose** | **WHO grade** | **Class** |
| --- | --- | --- | --- | --- | --- |
| Patient 1 | Female | 29 | Diffuse astrocytoma in the left frontal lobe; Secondary epilepsy | WHO grade II | LGG |
| Patient 2 | Male | 72 | Glioblastoma in the left temporal lobe; Secondary epilepsy | WHO grade IV | GBM |
| Patient 3 | Male | 37 | Embryonal tumors of the central nervous system in the left temporal lobe | WHO grade IV | GBM |
| Patient 4 | Male | 56 | Diffuse astrocytoma in the left frontal lobe | WHO grade II | LGG |

**Supplementary Table 1. Clinical information of donors enrolled in this study.**
