# Supplementary material for: Chromatin accessibility derived from cfDNA serves as a novel classification biomarker of glioma
Source: Front Oncol. 2025 Dec 15;15:1688625. doi: 10.3389/fonc.2025.1688625 (PMC12745158; doi:10.3389/fonc.2025.1688625)
Supplement: Supplementary Table 2 — The barcode adaptor sequence in Tn5-SALP-seq. [file Table2.docx]

**Supplementary Table 2. The barcode adaptor sequence in Tn5-SALP-seq**

| **Barcode ID** | **Adaptor sequence** | **Barcode** | **Match patient ID** |
| --- | --- | --- | --- |
| Barcode1 | GACTGGAGTTCAGACGTGTGCTCTTCCGATCTCTTGTAAGATGTGTATAAGAGACAG | CTTGTA | Patient 1 |
| Barcode2 | GACTGGAGTTCAGACGTGTGCTCTTCCGATCTACTTGAAGATGTGTATAAGAGACAG | ACTTGA | Patient 2 |
| Barcode3 | GACTGGAGTTCAGACGTGTGCTCTTCCGATCTCGATGTAGATGTGTATAAGAGACAG | CGATGT | Patient 3 |
| Barcode4 | GACTGGAGTTCAGACGTGTGCTCTTCCGATCTTAGCTTAGATGTGTATAAGAGACAG | TAGCTT | Patient 4 |
