# Supplementary material for: Chromatin accessibility derived from cfDNA serves as a novel classification biomarker of glioma
Source: Front Oncol. 2025 Dec 15;15:1688625. doi: 10.3389/fonc.2025.1688625 (PMC12745158; doi:10.3389/fonc.2025.1688625)
Supplement: Supplementary Table 3 — The mappable ration and reads number. [file Table3.docx]

**Supplementary Table 3. The mappable ration and reads number**

| **Patient ID** | **Sample type** | **Reads count** | **Mappable ratio** |
| --- | --- | --- | --- |
| Patient 1 | Tumor tissue genome DNA | 21504728 | 96.73% |
|  | Cerebrospinal fluid cfDNA | 6344428 | 95.78% |
|  | Plasma cfDNA | 40786371 | 40.76% |
| Patient 2 | Tumor tissue genome DNA | 471190 | 94.78% |
|  | Cerebrospinal fluid cfDNA | 18344197 | 94.76% |
|  | Plasma cfDNA | 16102680 | 70.80% |
| Patient 3 | Tumor tissue genome DNA | 9512907 | 97.16% |
|  | Cerebrospinal fluid cfDNA | 9796706 | 77.77% |
|  | Plasma cfDNA | 537042 | 86.44% |
| Patient 4 | Tumor tissue genome DNA | 22028619 | 97.04% |
|  | Cerebrospinal fluid cfDNA | 11900477 | 96.41% |
|  | Plasma cfDNA | 47197527 | 79.94% |
