# Supplementary material for: Chromatin accessibility derived from cfDNA serves as a novel classification biomarker of glioma
Source: Front Oncol. 2025 Dec 15;15:1688625. doi: 10.3389/fonc.2025.1688625 (PMC12745158; doi:10.3389/fonc.2025.1688625)
Supplement: Supplementary Table 5 — The sequence of primers used for glioma subtypes specific genome regions amplification. [file Table5.docx]

| Genome region | PCR primer | | Product length | Product format |
| --- | --- | --- | --- | --- |
|  | Forward | Reverse |  |  |
| chr8:699875-700375 target | CACAGGCCCTCACAGGCG | CCTGTGCGGGTCTGTGCG | - | Smearing* |
| chr10:128464235-128464735 target | TCAGAACAGGGACAGAGC | AAGTCAGGGTACGTGGGT | - | Non-specific bands* |
| chr1:236097067-236097567 target | GCTGAGGAGAACGAGGAA | GGTGTTGAGACCCGTATTT | - | Non-specific bands* |
| chr20:4136016-4136516 target | GGAACAGTAGCCCAAGACC | ACAGGAAGGAAACGCTCAAC | 201bp | Specific band |
| chr22:37328697-37329197 target | GGCTGCTGTGAGGTTAGA | GGGGAGCTGTAGGTCTGC | 136bp | Specific band |
| chr16:29604152-29604652 target | CTGGGTGGCTTGGTTTGC | CCAGTCAGCCAAGACAGCC | - | Smearing* |
| chr6:33491473-33491973 target | CTATGCTGCTCCATTCACTC | GCGTCTTAGCTTGGGTTG | 114bp | Specific band |

**Supplementary Table 5. The sequence of primers used for glioma subtypes specific genome regions amplification.**

*: The genome region contains repeat element. No specific primers can be designed.
